# Supplementary material for: Carbon-coated MoS1.5Te0.5 nanocables for efficient sodium-ion storage in non-aqueous dual-ion batteries
Source: Nat Commun. 2022 Feb 3;13:663. doi: 10.1038/s41467-022-28176-0 (PMC8814252; doi:10.1038/s41467-022-28176-0)
Supplement: Supplementary file 1 — Supplementary information [file 41467_2022_28176_MOESM1_ESM.pdf]

Supplementary information

Carbon-coated $MoS_{1.5}Te_{0.5}$ nanocables for efficient sodium-ion storage in non-aqueous dual-ion batteries

Yangjie Liu,[1,2] Xiang Hu,[1,2] Junwei Li,[2] Guobao Zhong,[1,2] Jun Yuan,[1,2] Hongbing Zhan,[1,*] Yongbing Tang,[3] Zhenhai Wen[2,*]

[1]*College of Materials Science and Engineering, Fuzhou University, Fuzhou 350108, P. R. China.*

[2]*CAS Key Laboratory of Design and Assembly of Functional Nanostructures, and Fujian Provincial Key Laboratory of Nanomaterials, Fujian Institute of Research on the Structure of Matter, Chinese Academy of Sciences, Fuzhou, Fujian 350002, P. R. China.*

[3]*Functional Thin Films Research Center, Shenzhen Institutes of Advanced Technology, Chinese Acadmy of Sciences, Shenzhen 518055, P. R. China.*

** Corresponding authors*
E-mail: hbzhan@fzu.edu.cn, wen@fjirsm.ac.cn

Supplementary Figures

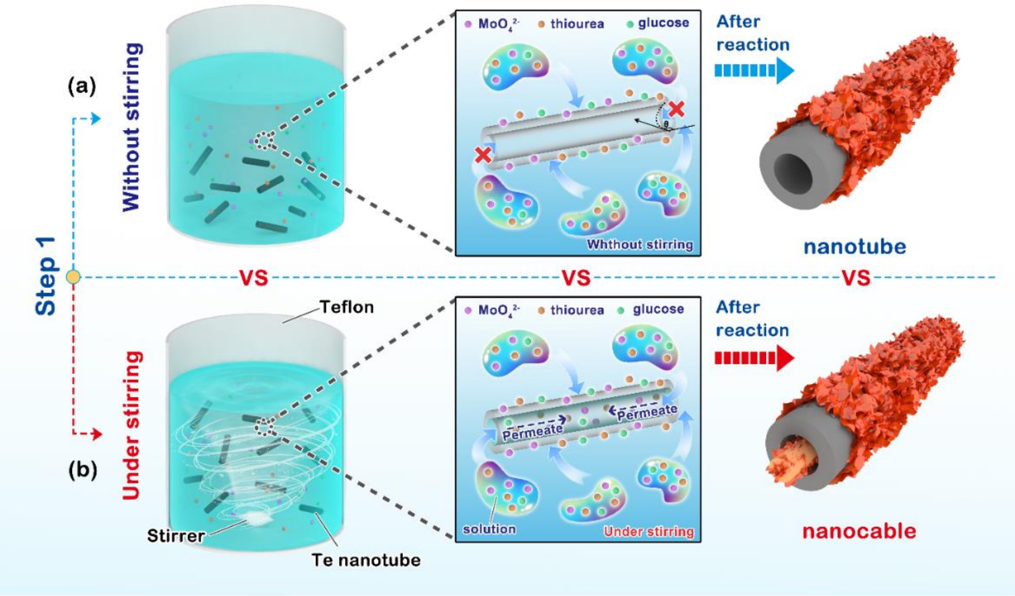

Supplementary Figure 1. Schematic illustration of the formation process of nanotube and nanocable structures under normal and stirring hydrothermal processes (Step 1) at 180 °C for 10 h respectively.

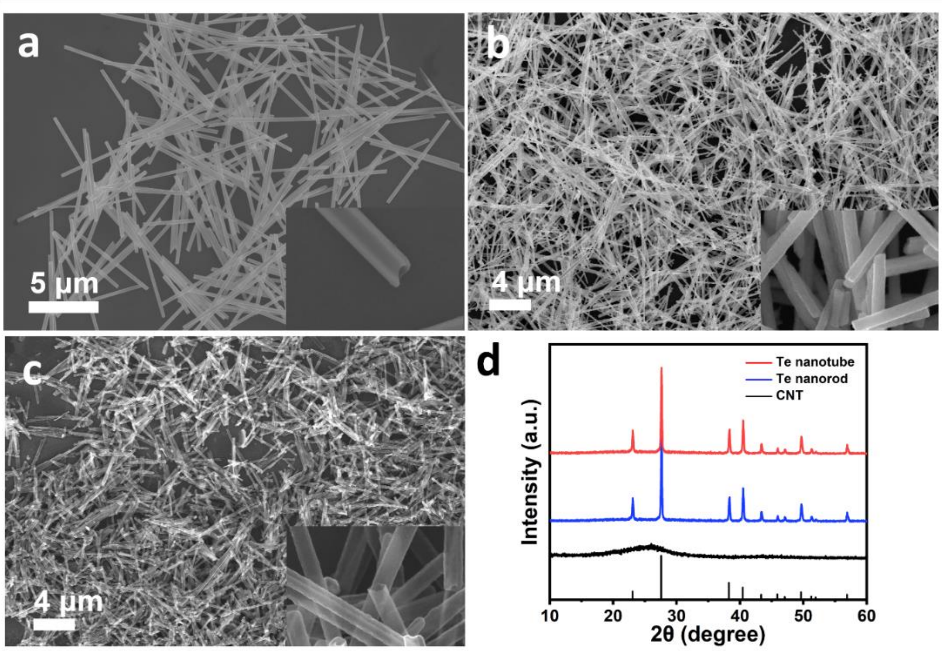

Supplementary Figure 2. SEM images of (a) Te nanotubes, (d) Te nanorods and (c) CNT and corresponding XRD pattern.

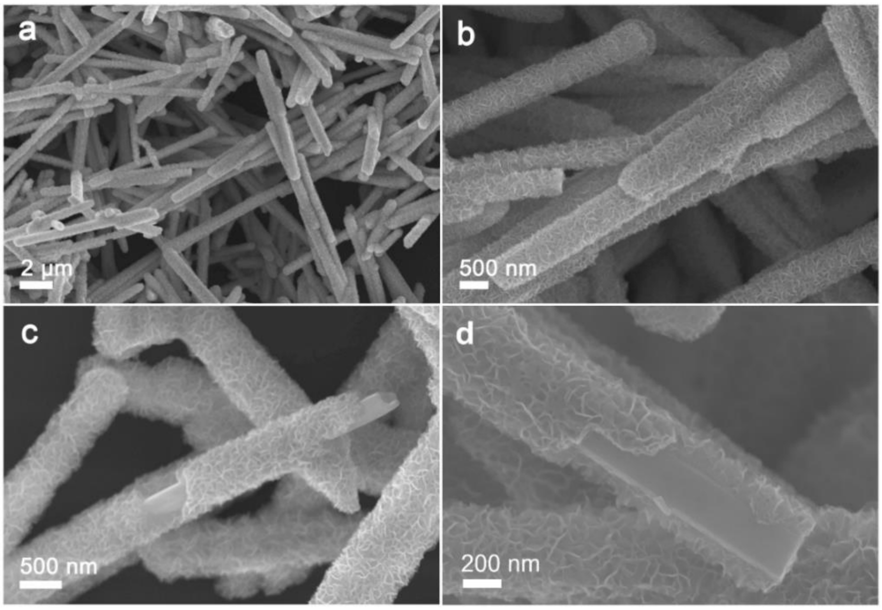

Supplementary Figure 3. (a-c) SEM images of Te@C/MoS_2.

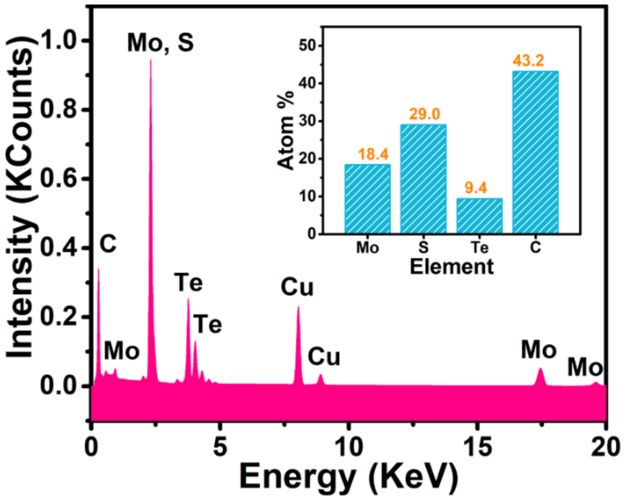

Supplementary Figure 4. EDS spectrum and table (inset) illustrating the element content contained in $MoS_{1.5}Te_{0.5}$@C nanocables.

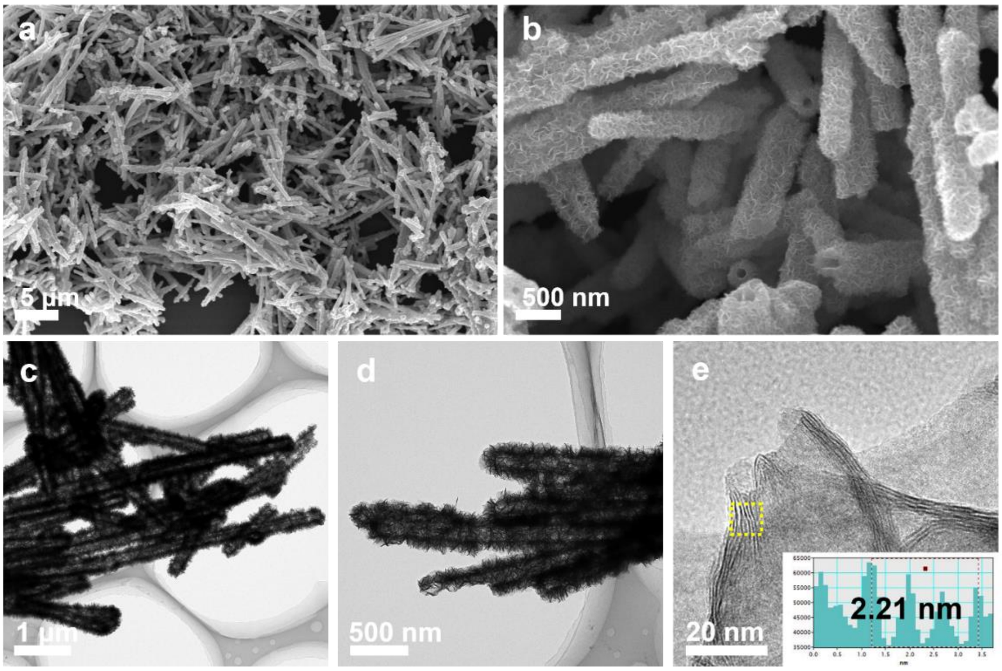

Supplementary Figure 5. (a-b) SEM images and (c-e) TEM images of $MoS_{1.5}Te_{0.5}$@C nanotubes.

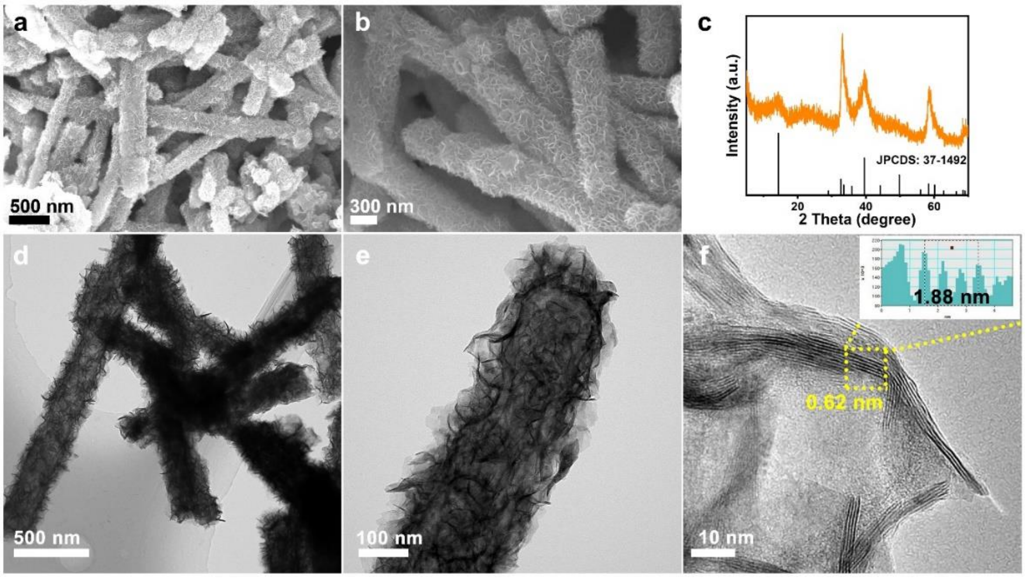

Supplementary Figure 6. (a,b) SEM images, (c) XRD pattern and (d-f) TEM images of CNT@MoS_2 nanotubes.

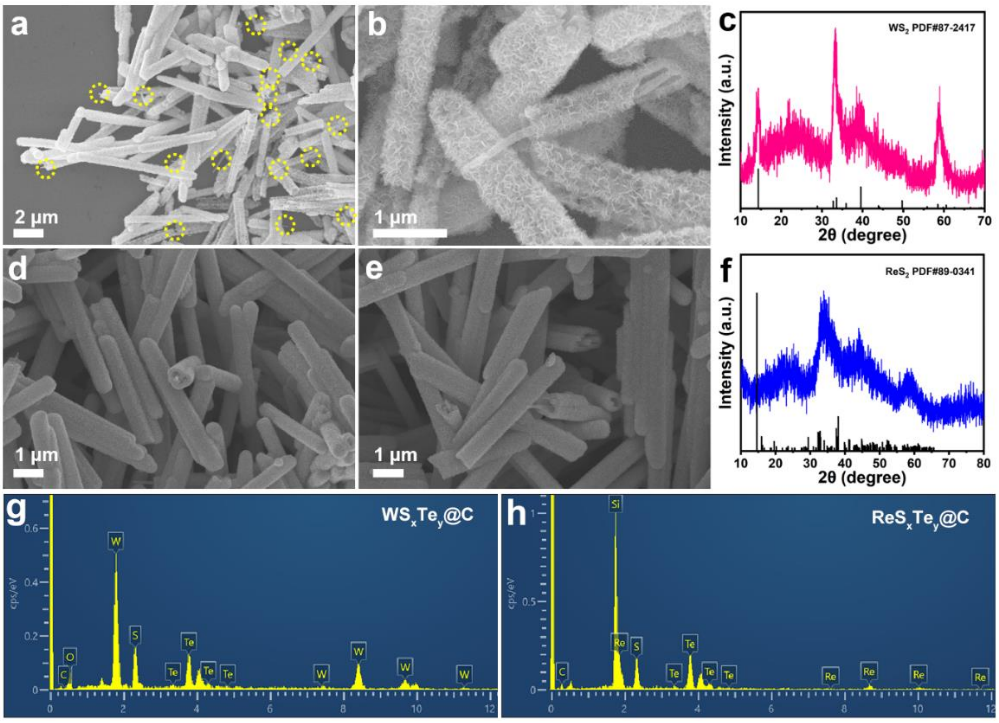

Supplementary Figure 7. The SEM images of (a,b) wire-in-tube WS_xTe_y@C nanocables and (d,e) wire-in-tube ReS_xTe_y@C nanocables. XRD results of (c) wire-in-tube WS_xTe_y@C nanocables and (f) wire-in-tube ReS_xTe_y@C nanocables. EDX spectrum of the WS_xTe_y@C nanocables (g) and ReS_xTe_y@C nanocables (h).

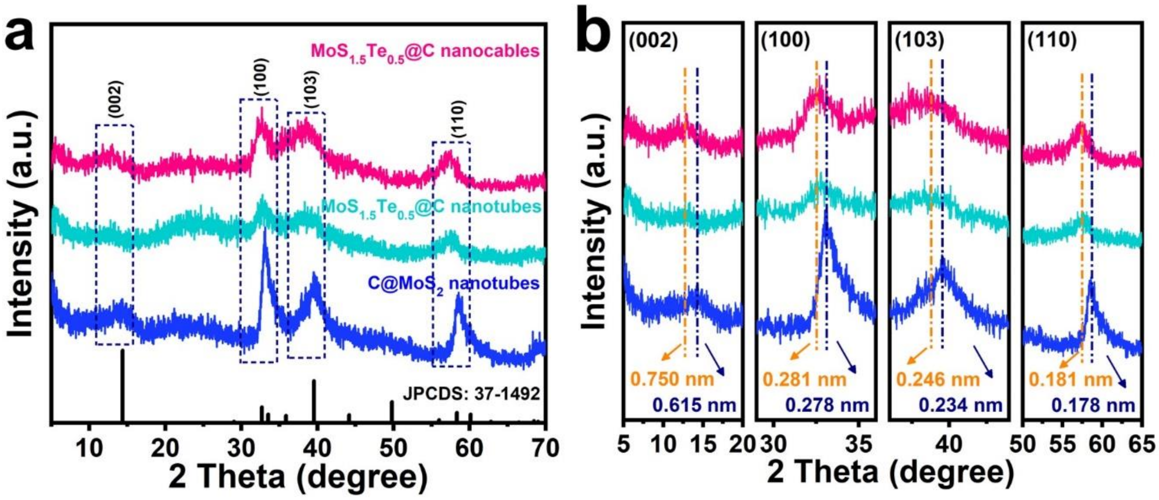

Supplementary Figure 8. XRD patterns of $MoS_{1.5}Te_{0.5}$@C nanocables, $MoS_{1.5}Te_{0.5}$@C nanotubes, and CNT@MoS_2 nanotubes composites.

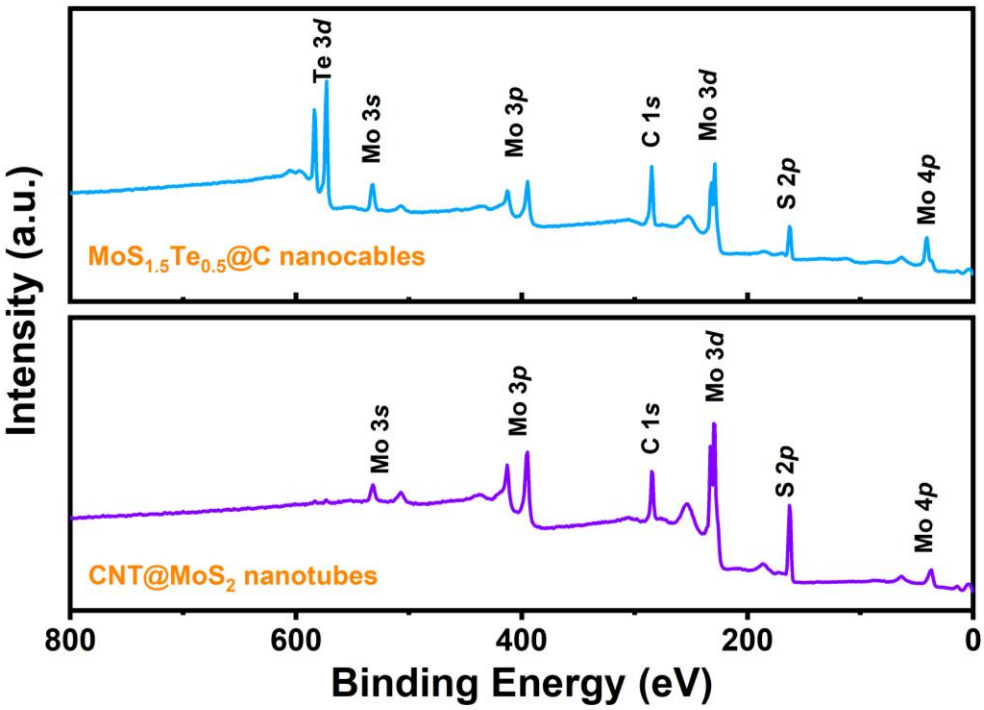

Supplementary Figure 9. The survey XPS spectrum of the $MoS_{1.5}Te_{0.5}$@C nanocables and the CNT@MoS_2 composites.

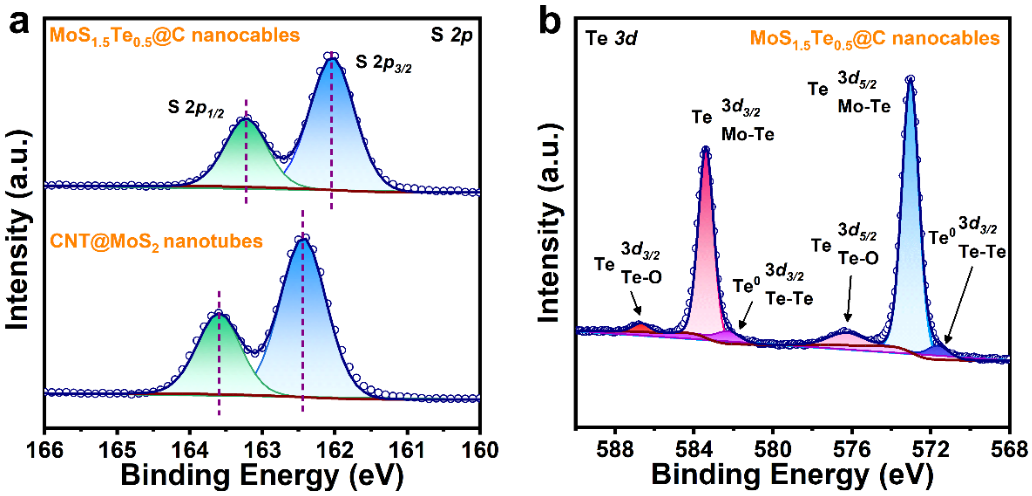

Supplementary Figure 10. High-resolution XPS spectra for (a) S 2*p* and (b) Te 3*d* of $MoS_{1.5}Te_{0.5}$@C nanocables composites.

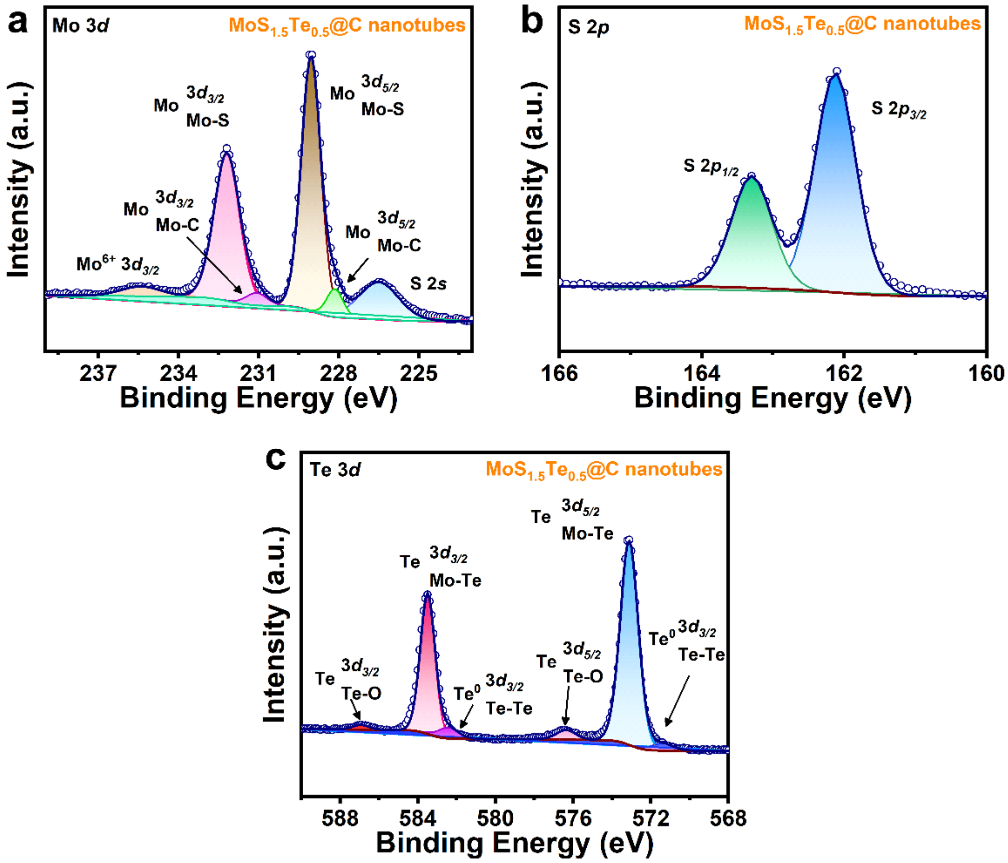

Supplementary Figure 11. High-resolution XPS spectra for (a) Mo 3*d*, (b) S 2*p* and (c) Te 3*d* of $MoS_{1.5}Te_{0.5}$@C nanotubes composites.

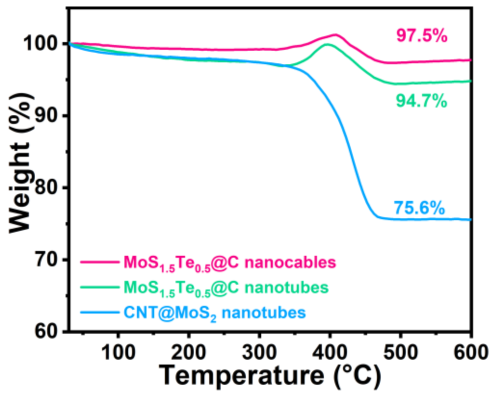

Supplementary Figure 12. TGA curves of $MoS_{1.5}Te_{0.5}$@C nanocables, $MoS_{1.5}Te_{0.5}$@C nanotubes and CNT@MoS_2 nanotubes samples under air atmosphere.

An initial weight loss below 200 °C is attributed to the evaporation and desorption of surface adsorbed water molecules. The second weight loss between 200-700 ºC is the oxidation of active substance $MoS_{1.5}Te_{0.5}$ in the composite into MoO_3, SO_2, and TeO_2, and the carbon is transformed into CO_2. It's worth noting that the weight increase in 200-400 ºC is attributed with the generation of TeO_2. Form the above, the residual weight percent of $MoS_{1.5}Te_{0.5}$@C nanocables, $MoS_{1.5}Te_{0.5}$@C nanotubes and CNT@MoS_2 nanotubes samples at 650 °C are 97.5%, 94.7% and 75.6%, respectively. The content of $MoS_{1.5}Te_{0.5}$ (ω_{MoSTe}) in the $MoS_{1.5}Te_{0.5}$@C nanocables and $MoS_{1.5}Te_{0.5}$@C nanotubes samples are calculated from the Supplementary Equation (1), and the content of MoS_2 (ω_{MoS2}) in the CNT@MoS_2 nanotubes also could be obtained from the Supplementary Equation (2):

$$\omega_{MoS_{1.5}Te_{0.5}} = \frac{M_{MoS_{1.5}Te_{0.5}}}{M_{(MoO_3 + TeO_2)}} * \omega_{(MoO_3 + TeO_2)} \quad (1)$$

$$\omega_{MoS_2} = \frac{M_{MoS_2}}{M_{MoO_3}} * \omega_{(MoO_3)} \quad (2)$$

$$\omega_{Te} = \frac{M_{Te}}{M_{MoS_{1.5}Te_{0.5}}} * \omega_{MoS_{1.5}Te_{0.5}} \quad (3)$$

Thus, the active substance content in $MoS_{1.5}Te_{0.5}$@C nanocables, $MoS_{1.5}Te_{0.5}$@C nanotubes and CNT@MoS_2 nanotubes samples are calculated at 90.9 %, 87.9 % and 84.1 %, respectively. On the other hand, the corresponding carbon content in the three samples is 9.1 %, 12.1 %, and 15.9 %, respectively. Impressively, the Te atoms doping with a high loading (~30 wt%) in the $MoS_{1.5}Te_{0.5}$@C nanocables could be achieved (Supplementary Equation (3)).

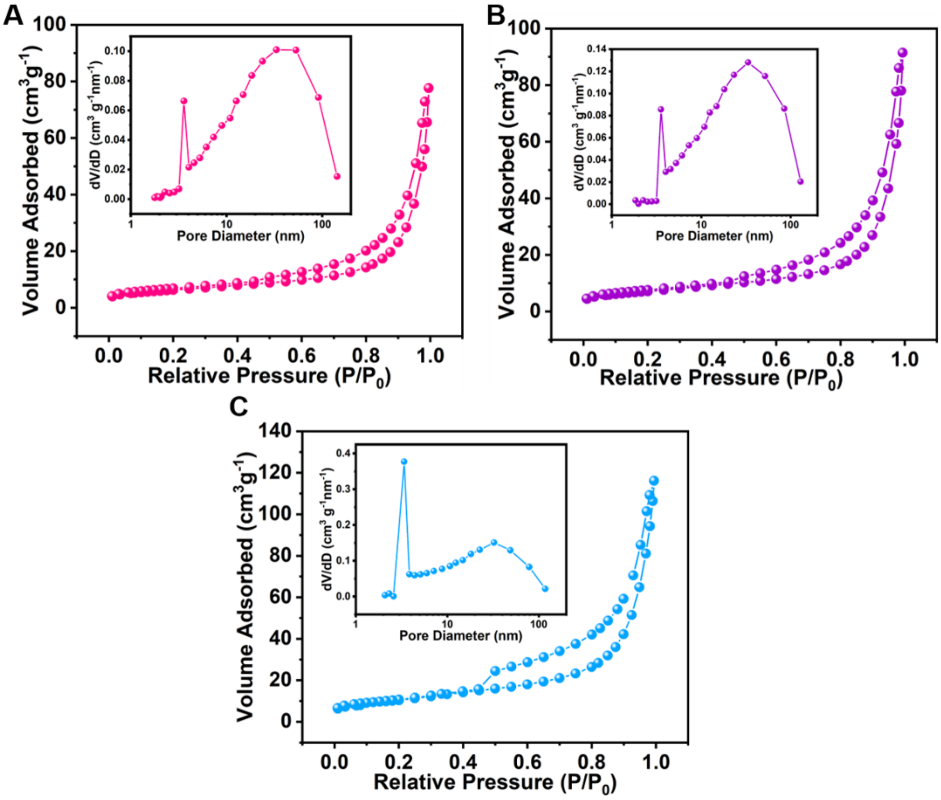

Supplementary Figure 13. Nitrogen adsorption-desorption isothermal curves (inset: pore size distribution) for (a) $MoS_{1.5}Te_{0.5}$@C nanocables, (b) $MoS_{1.5}Te_{0.5}$@C nanotubes and (c) CNT@MoS_2 nanotubes.

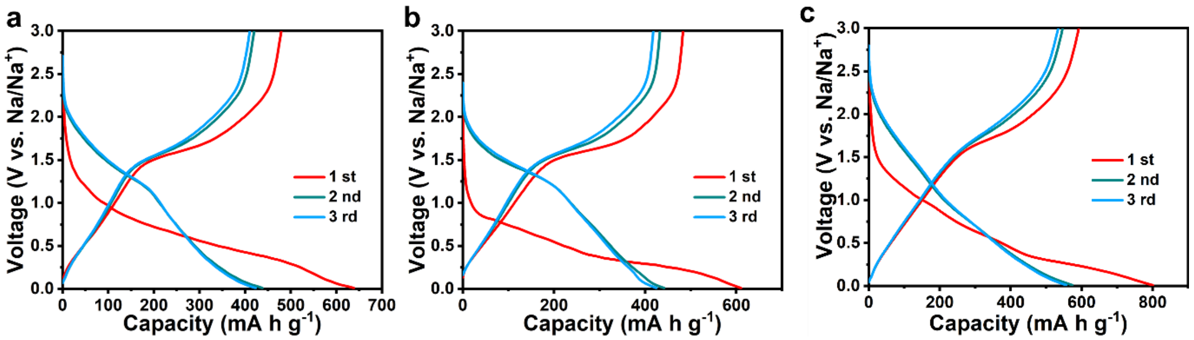

Supplementary Figure 14. Charge-discharge profiles of (a) the $MoS_{1.5}Te_{0.5}$@C nanocables, (b) the $MoS_{1.5}Te_{0.5}$@C nanotubes and (c) the CNT@MoS_2 electrodes tested in combination with Na metal electrode at a specific current of 0.1 A g^{-1}. The electrochemical measurements were carried out using a 1 M $NaPF_6$ in EC/EMC/DMC (1:1:1, by volume) electrolyte solution at 25 °C.

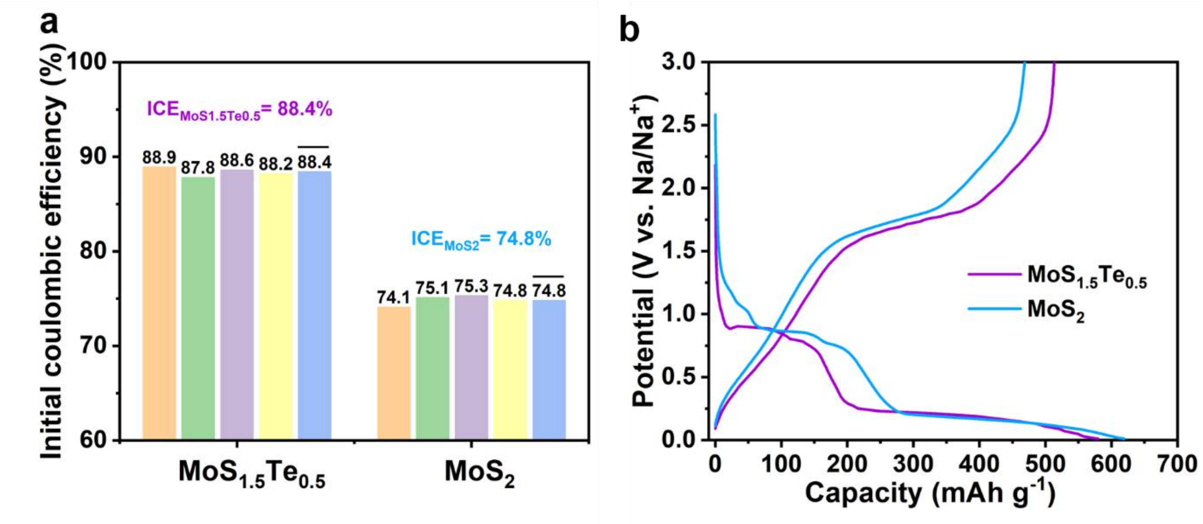

Supplementary Figure 15. a) ICE for two different set of electrochemical experiments comprising five different Na metal||$MoS_{1.5}Te_{0.5}$ and Na metal||MoS_2 cells each and, b) the 1st sodiation/desodiation potential profiles of the $MoS_{1.5}Te_{0.5}$- and MoS_2-based electrodes. The electrochemical measurements were carried out using a 1 M $NaPF_6$ in EC/EMC/DMC (1:1:1, by volume) electrolyte solution at 100 mA g^{-1} and 25 °C.

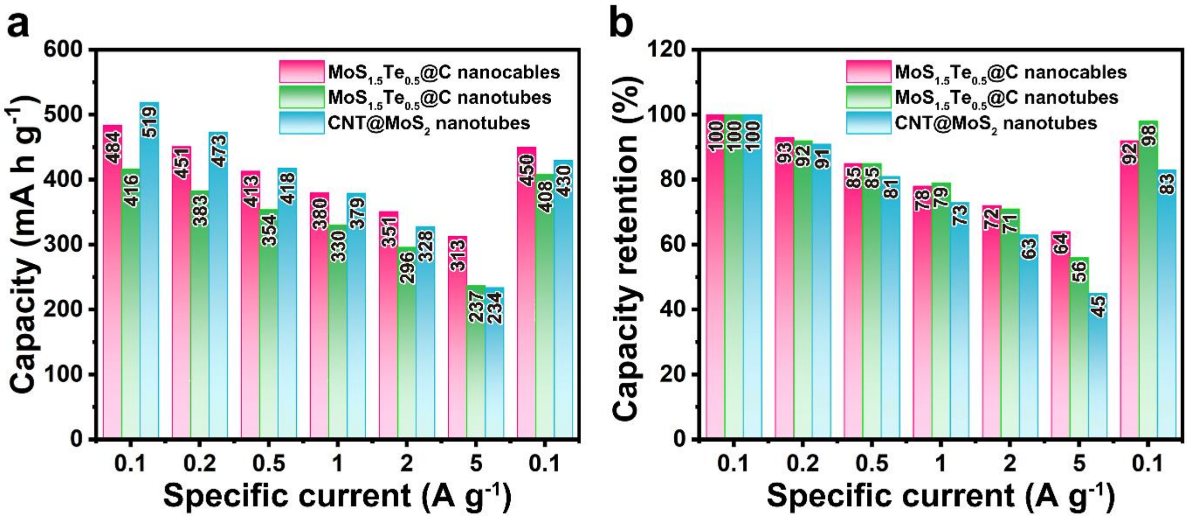

Supplementary Figure 16. (a) The average capacities and (b) the capacity retention rate of the $MoS_{1.5}Te_{0.5}$@C nanocables, $MoS_{1.5}Te_{0.5}$@C nanotubes and CNT@MoS_2 nanotubes electrodes sampled at the 5th discharge cycles for Na metal half-cell testing at various current rates based on the data used for Figure 3d.

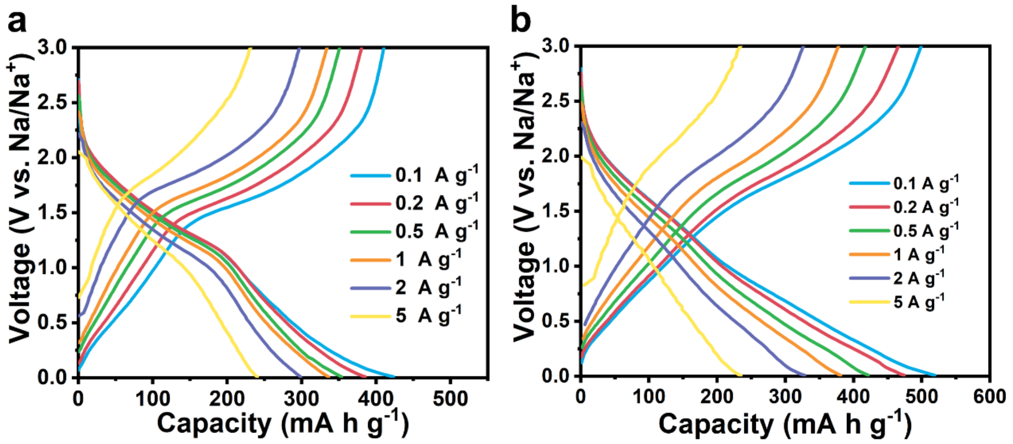

Supplementary Figure 17. Charge-discharge profiles of (a) the $MoS_{1.5}Te_{0.5}$@C nanotubes and (b) the CNT@MoS_2 electrodes tested in combination with a Na metal counter electrode at various specific currents (from 0.1 to 5.0 A g^{-1}). The electrochemical measurements were carried out using a 1 M $NaPF_6$ in EC/EMC/DMC (1:1:1, by volume) electrolyte solution at 25 °C.

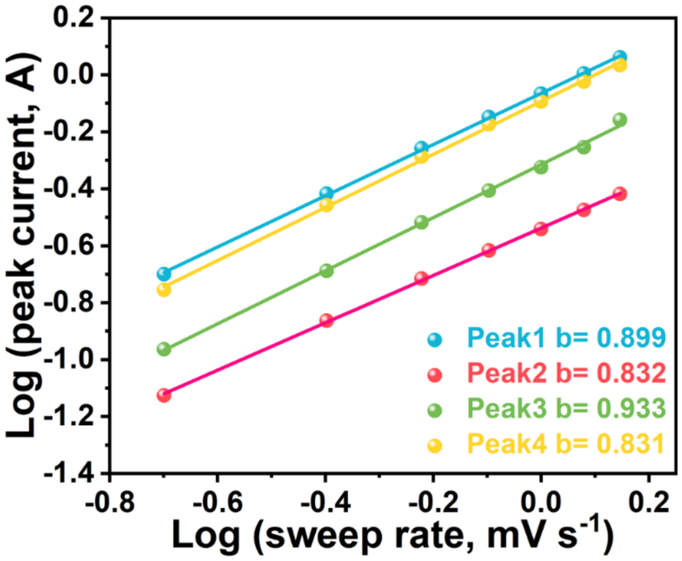

Supplementary Figure 18. Log (i) versus log (v) plots at different redox peaks of the $MoS_{1.5}Te_{0.5}$@C nanocables.

The calculated b-value for $MoS_{1.5}Te_{0.5}$@C nanocables electrode based on the linear fitting of log(v) against log(i) are 0.89, 0.83, 0.93 and 0.83 in the corresponding redox peaks, respectively, indicating the Na^+ storage behavior in $MoS_{1.5}Te_{0.5}$@C nanocables electrode is mainly dominated by a capacitive charge storage behavior.

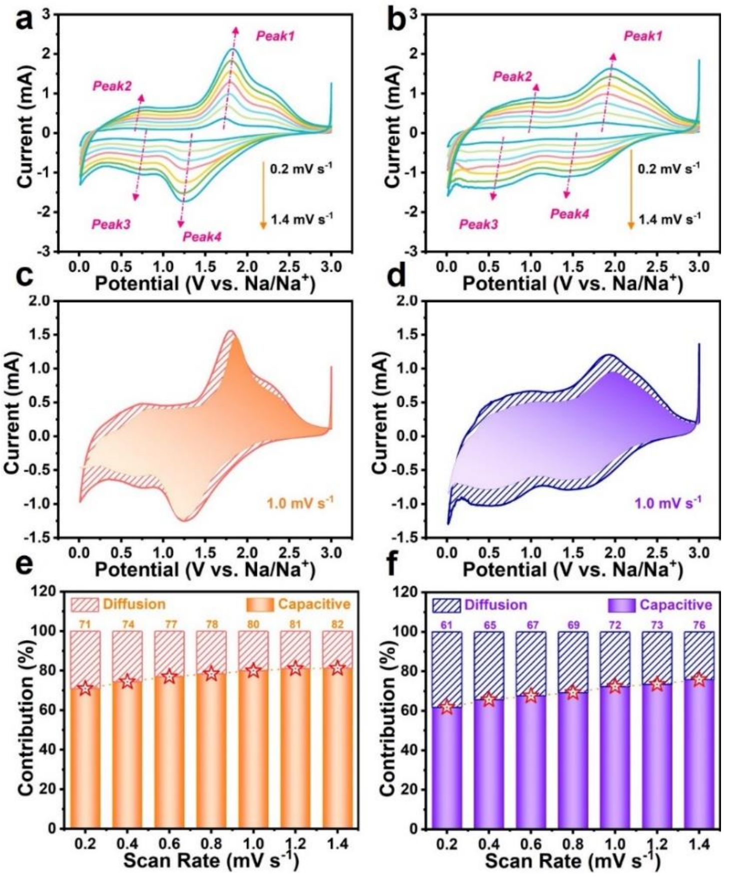

Supplementary Figure 19. (a,b) CV curves at various scan rates, (c,d) Capacitive contribution at 1.0 mV s^{-1}, (e,f) the percentages of capacitive and diffusion-controlled capacities at different scan rates of the $MoS_{1.5}Te_{0.5}$@C nanotubes and the CNT@MoS_2 respectively.

As shown in Figure 3f, Supplementary Figure 19, one can obverse that the $MoS_{1.5}Te_{0.5}$@C nanocables show a more positive cathodic peak and a more negative anodic peak, as well as enhanced peaks intensity than that of the CNT@MoS_2 nanotubes electrode, implying the accelerated conversion reaction kinetics and dramatically decreased polarization of liquid-solid conversion by Te-doping. Such significantly enhanced reaction kinetics could be ascribed to these following factors: (1) The anionic Te doping to form $S_{1.5}Te_{0.5}$ interlayer ligands narrow interlayer energy band of MoS_2 and thus achieve advanced metallic properties, improve the electrical conductivity of materials. (2) The weaken Mo-Te interaction is easier to break than that of Mo-S bands, leading to the reversible conversion reaction dynamics and reversibility.

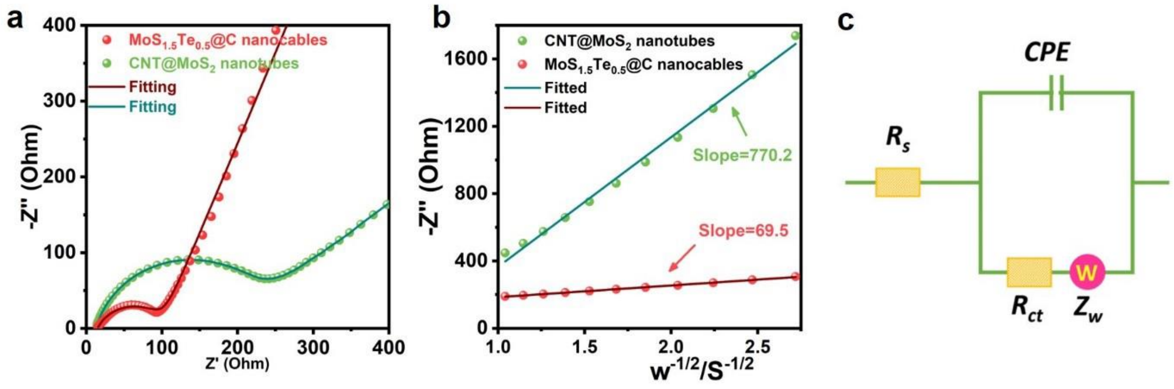

Supplementary Figure 20. (a) Electrochemical impedance spectra (EIS) of the $MoS_{1.5}Te_{0.5}$@C nanocables and the CNT@MoS_2 electrodes and (b) linear fits of the Z' versus $\omega^{-1/2}$ ($\omega = 2\pi f$) in the low-frequency region. (c) the corresponding equivalent circuit model.

The Na^+ diffusion coefficient (D_{Na+}) in $MoS_{1.5}Te_{0.5}$@C nanocables electrode is higher than these in the CNT@MoS_2 nanotubes electrodes basing on the slope of the oblique line in Supplementary Figure 20b.

The equivalent circuit of EIS spectra was modeled to fit the Nyquist plots, where R_s, R_{ct} and R_f represent the total ohmic resistance of the electrode system, charge-transfer resistance in the middle-frequency region and SEI films resistance in the high-frequency region, respectively. Z_w is the Warburg impedance for Na^+ diffusion in the bulk of the electrode materials associated with the straight sloping line at low frequency region. CPE related to the surface capacitance.

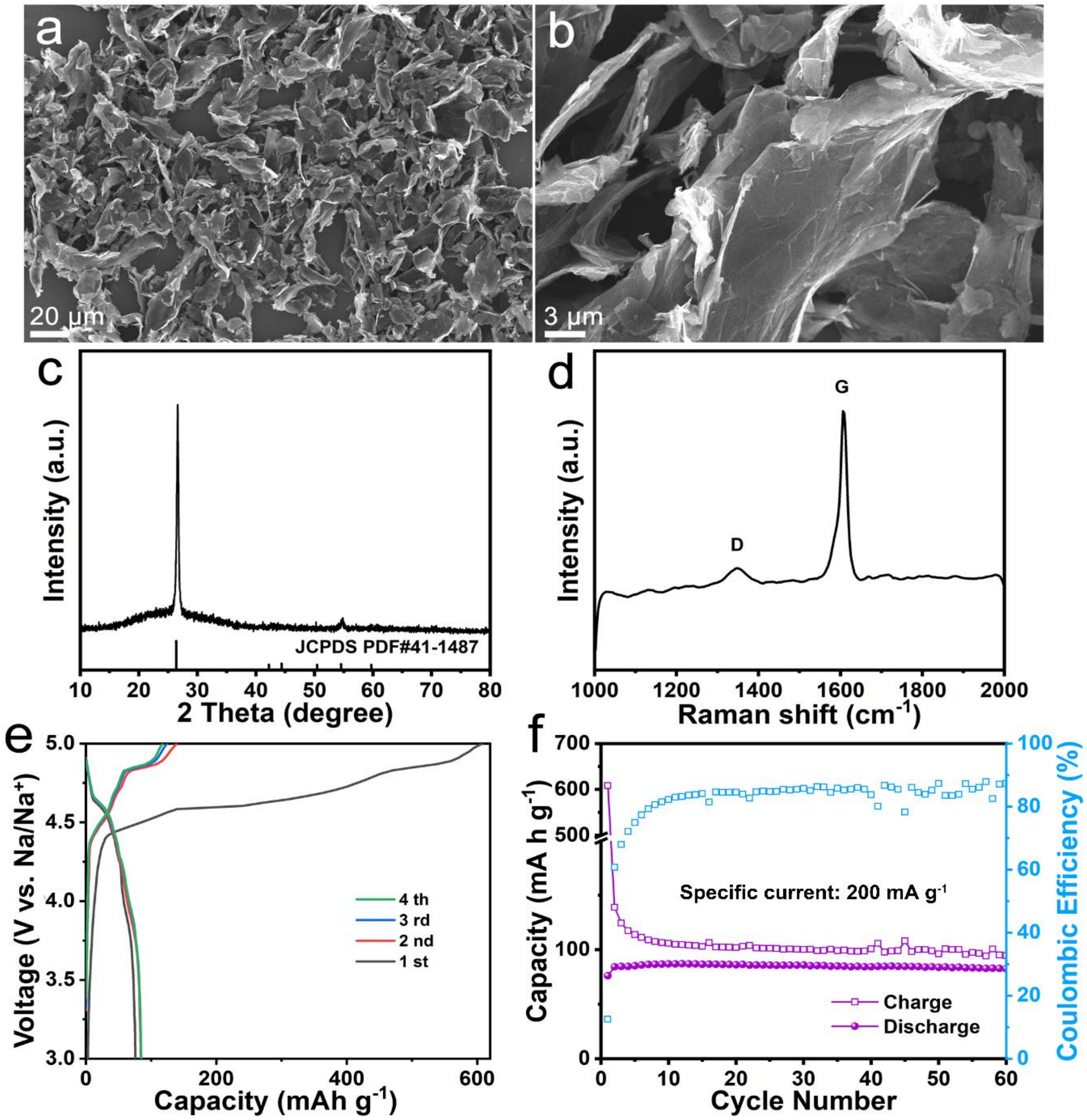

Supplementary Figure 21. (a, b) FE-SEM, (c) XRD peaks, (d) Raman spectrum of EG. (e) Charge-discharge curves at the initial three cycles and (f) Cycling performance under 200 mA g^{-1} for the Na metal||EG cell. The electrochemical measurements were carried out using a 3 M $NaPF_6$ in EC/EMC/DMC (1:1:1, by volume) electrolyte solution at 25 °C.

For the anion-hosting cathodes, the low CE of EG cathode could be attributed to the partially irreversibly side reactions occurs during the anion intercalation reaction with the large size of anions and the decomposition of electrolyte at a high voltage.

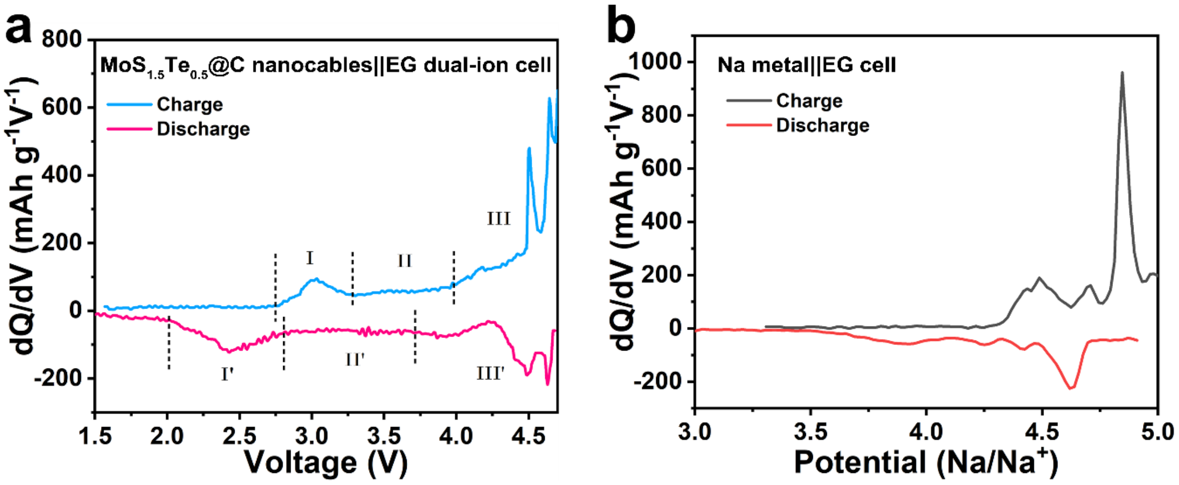

Supplementary Figure 22. The dQ/dV differential curve of (a) the $MoS_{1.5}Te_{0.5}$@C nanocables||EG dual-ion cell and (b) Na metal||EG cell.

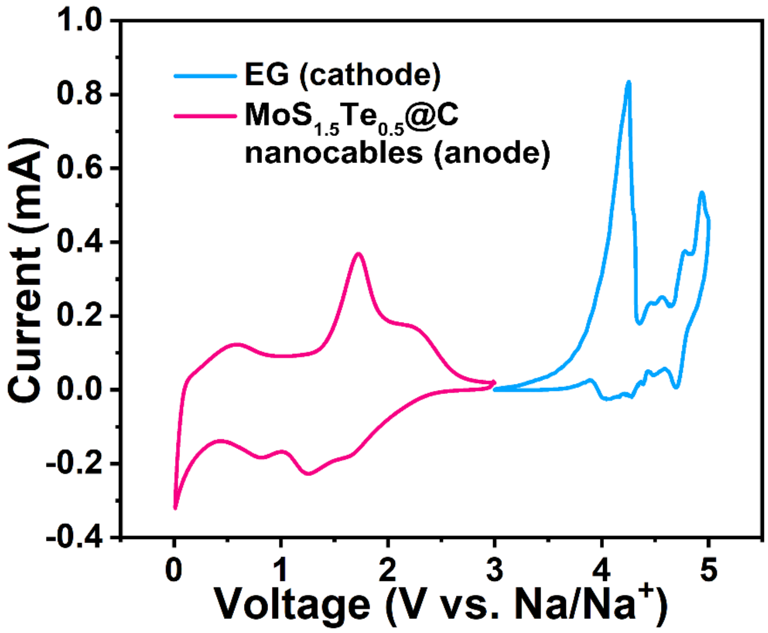

Supplementary Figure 23. The CV curves of the $MoS_{1.5}Te_{0.5}$@C nanocables and EG half-cells with the device configuration of Na metal||$MoS_{1.5}Te_{0.5}$@C nanocables and Na metal||EG cells at a scan rate of 0.1 mV s^{-1}.

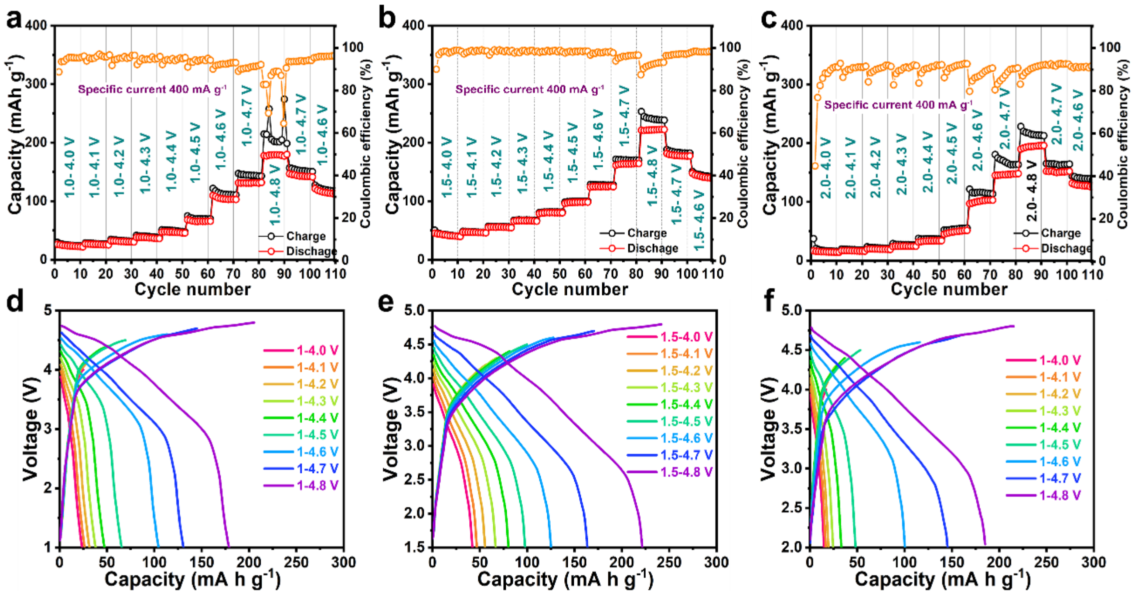

Supplementary Figure 24. The cycling behaviour of various $MoS_{1.5}Te_{0.5}$@C nanocables||EG dual-ion cells with same charge voltage cut-off from 4.0 to 4.8 V and various discharge voltage cut-off under 1.0 V (a), 1.5 V (b), and 2.0 V (c). (d-f) corresponding charge-discharge curves at different voltage windows. The electrochemical measurements were carried out using a 1 M $NaPF_6$ in EC/EMC/DMC (1:1:1, by volume) electrolyte solution at 25 °C.

In order to legitimately explore the suitable voltage window in this SDIB system, various charge cut-off voltages are paired with identical discharge cut-off voltage at 1.0 V, 1.5 V and 2.0 V, respectively (Supplementary Figure 24). It is noted that the SDIBs exhibit a much low capacity, when the charge cut-off voltage below 4.6 V. However, if the charge cut-off voltage exceeds 4.8 V, there is an obvious capacity gap between charge/discharge curves. In other words, the CE begins to decay owing to the side reaction, which could be attributed by the decomposition of electrolyte under high voltage. Thus, value of 4.7 V is chosen as the charge cut-off voltage due to its high CE and higher capacity. Therefore, we apply the fixed charge cut-off voltage of 4.7 V to match different discharge cut-off voltage. For the discharge cut-off voltage at 1.0 V, the SDIBs fail to get a satisfactory capacity; while for such value at 2.0 V, one can observe that the CE is almost below 90 % at various rates. Therefore, we select an optimized operating voltage windows range from 1.5 V to 4.7 V for subsequent test.

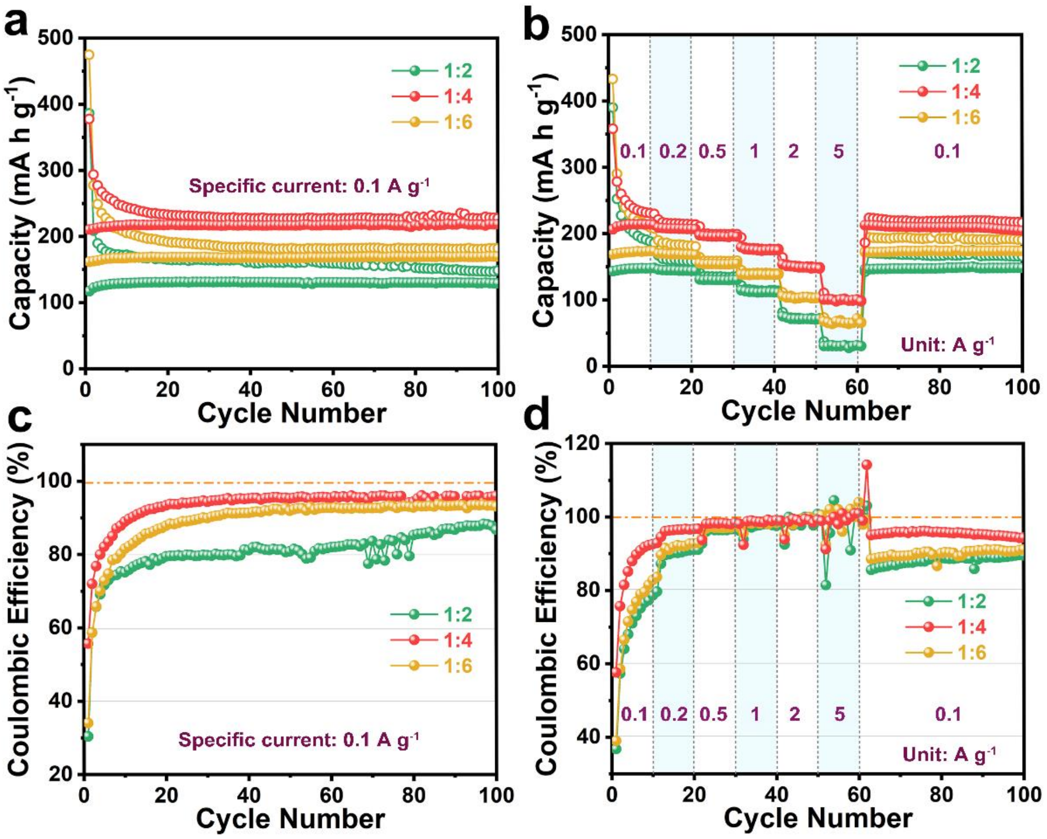

Supplementary Figure 25. (a) Cycling stability under specific current of 0.1 A g^{-1} and (b) rate performance profiles under various rates (from 0.1 A g^{-1} to 5 A g^{-1}) for $MoS_{1.5}Te_{0.5}$@C nanocables||EG dual-ion cells with different anode/cathode mass ratio (1:2, 1:4 and 1:6). Corresponding Coulombic efficiency for cycle performance (c) and rate capability (d). The electrochemical measurements were carried out using a 3 M $NaPF_6$ in EC/EMC/DMC (1:1:1, by volume) electrolyte solution at 25 °C.

To obtain the desired electrochemical performance, we must balance the capacity between cathode and anode. The $MoS_{1.5}Te_{0.5}$@C nanocables anode showed a specific capacity of around 450 mAh g^{-1}, and the graphite cathode exhibited a specific capacity of around 100 mAh g^{-1}, thus the mass ratio of the EG cathode to $MoS_{1.5}Te_{0.5}$@C nanocables anode was optimized to be 4:1 according to the charge balance Supplementary Equation (4):

$$C_{\text{cathode}} \times m_{\text{cathode}} = C_{\text{anode}} \times m_{\text{anode}} \quad (4)$$

where C (mAh g^{-1}) and m (mg) are the specific capacity of both electrodes and the mass of active materials, respectively. Meanwhile, we also tested the cells with different anode/cathode mass ratios (1:2, 1:4 and 1:6) in a 3 M $NaPF_6$ in EC/EMC/DMC (1:1:1, by volume) electrolyte solution.

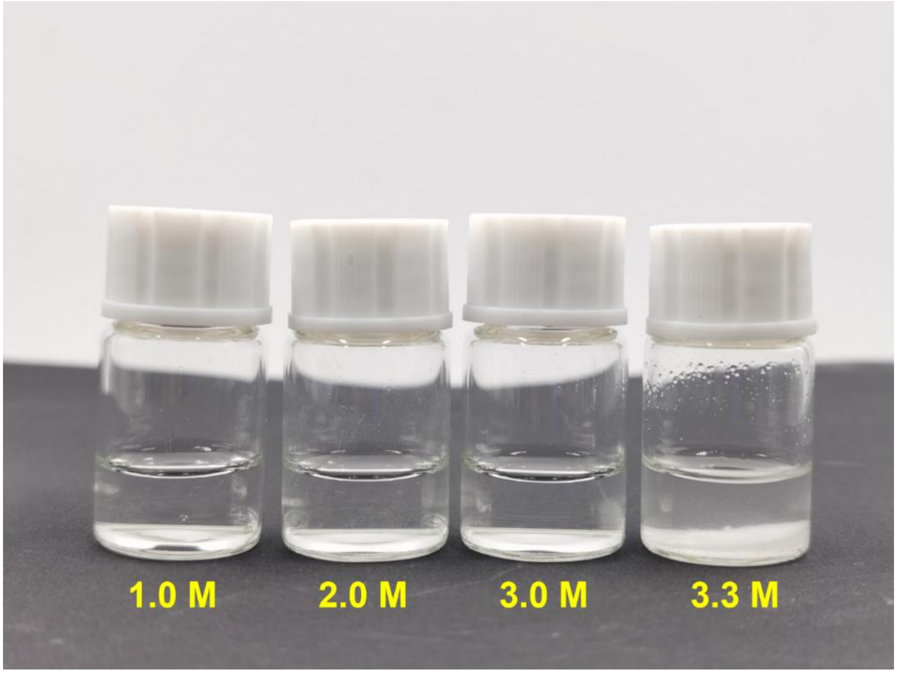

Supplementary Figure 26. The digital photographs with different concentration electrolytes. (1.0, 2.0, 3.0 and 3.3 M $NaPF_6$ in EC/DMC/DEC mixture (1:1:1, by volume)).

It should be noted that the solubility of $NaPF_6$ in alkyl carbonates is relatively limited at room temperature. When the concentration exceeds 3.3 M, the electrolyte solution is saturated, and a white precipitate can be noticed in the vial.

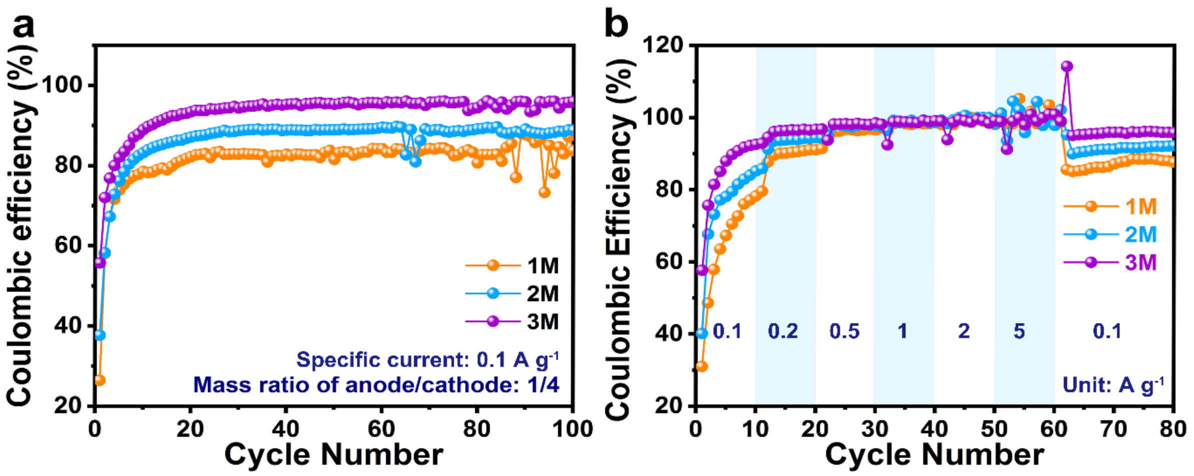

Supplementary Figure 27. Corresponding coulombic efficiency of the $MoS_{1.5}Te_{0.5}$@C nanocables||EG dual-ion cells for (a) cycling performance and (b) Rate performance using different electrolyte concentrations (i.e., 1.0, 2.0 and 3.0 M $NaPF_6$ in EC/DMC/DEC mixture (1:1:1, by volume)).

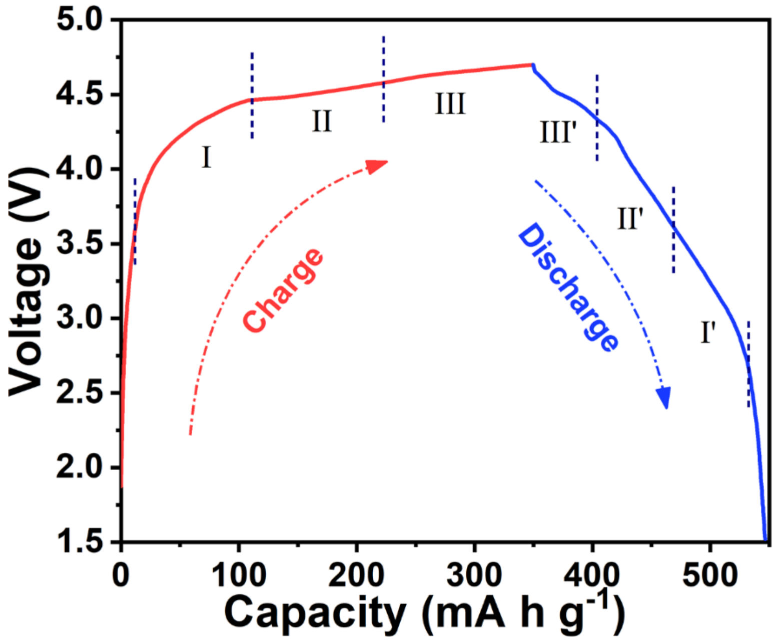

Supplementary Figure 28. 1st charge/discharge cycle of the $MoS_{1.5}Te_{0.5}$@C nanocables||EG dual-ion cell cycle at 0.1 A g^{-1} and 25 °C.

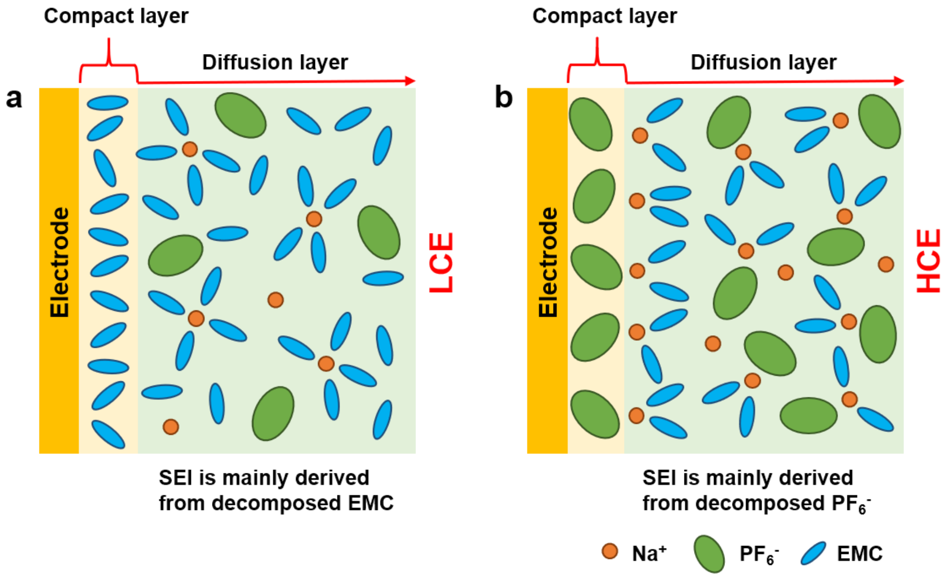

Supplementary Figure 29. The SEI layer formation mechanism in SDIBs using (a) low concentrated electrolyte and (b) high concentrated electrolyte.

The compositions of SEI layer in low concentrated electrolyte (LCE) and high concentrated electrolyte (HCE) are quite different (Supplementary Figure 29). In LCE, the SEI layer is mainly originated from the decomposition of solvents, which is loose and thermodynamically unstable, thus inevitably aggravating continuation of side reaction and decreasing the coulombic efficiency. However, the association between Na^+ ions and the anions in HCE can be enhanced by the high concentrated Na salts, reducing the presence of the free solvent molecules. Therefore, the SEI layer is mainly derived from by the anions of Na salts. This is thinner, more compact, and thermodynamically stable, which can effectively further suppress the decomposition of electrolyte and ensure high reversibility for ions intercalation/deintercalation, which is benefit for maintaining high coulombic efficiency and durable cycling performance of SDIBs.[1]

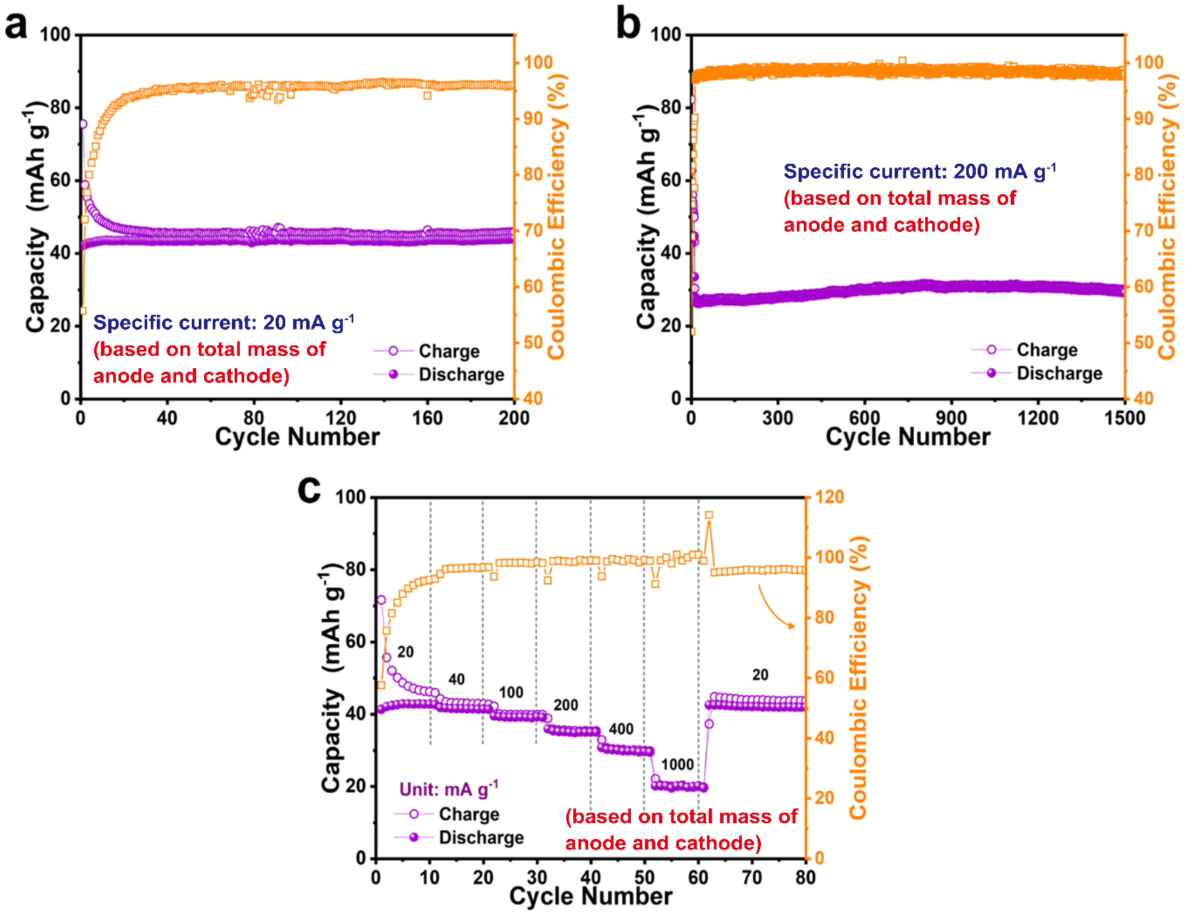

Supplementary Figure 30. The cycling performance at (a) 20 mA g^{-1} and (b) 200 mA g^{-1} and (c) the rate performance of the $MoS_{1.5}Te_{0.5}$@C nanocables||EG dual-ion cells (The specific capacity and specific current were calculated based on the total mass of the cathode and anode active materials).

The initial specific capacity of the $MoS_{1.5}Te_{0.5}$@C nanocables||EG dual-ion cell is about 216.8 mAh g^{-1} based on the anode material. By counting in the cathode material mass, which is about 4 times of the anode material mass, the capacity calculated based on total mass of cathode and anode is about 43.4 mAh g^{-1} (= 216.8 mAh g^{-1}/(1 + 4)).

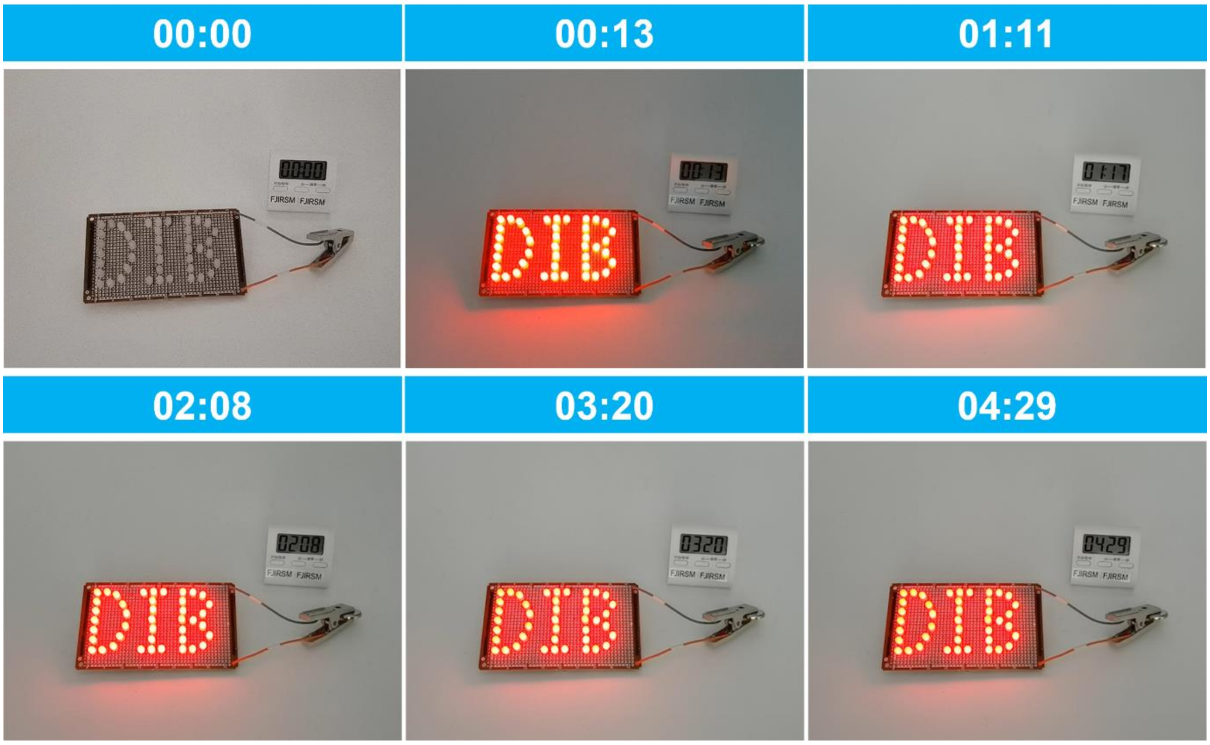

Supplementary Figure 31. Digital photographs of the “DIB” logo composing of 42 light-emitting-diodes (LEDs) light up by one single $MoS_{1.5}Te_{0.5}$@C nanocables||EG dual-ion cell at different times.

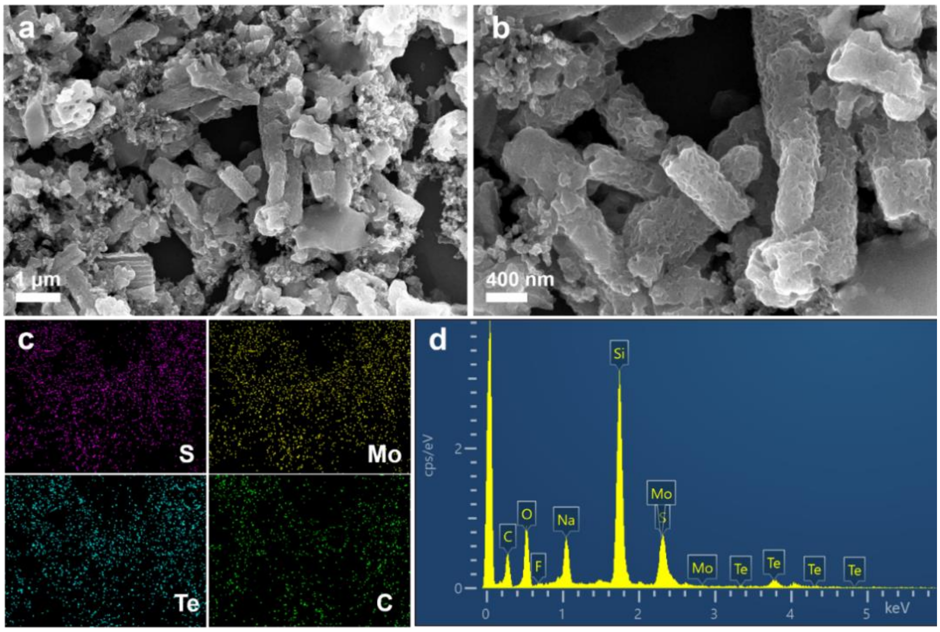

Supplementary Figure 32. (a,b) SEM images, (c) element mapping and (d) EDS analysis of a postmortem $MoS_{1.5}Te_{0.5}$@C nanocables electrode cycled 1500 times at 1 A g^{-1} in $MoS_{1.5}Te_{0.5}$@C nanocables||EG dual-ion cell configuration.

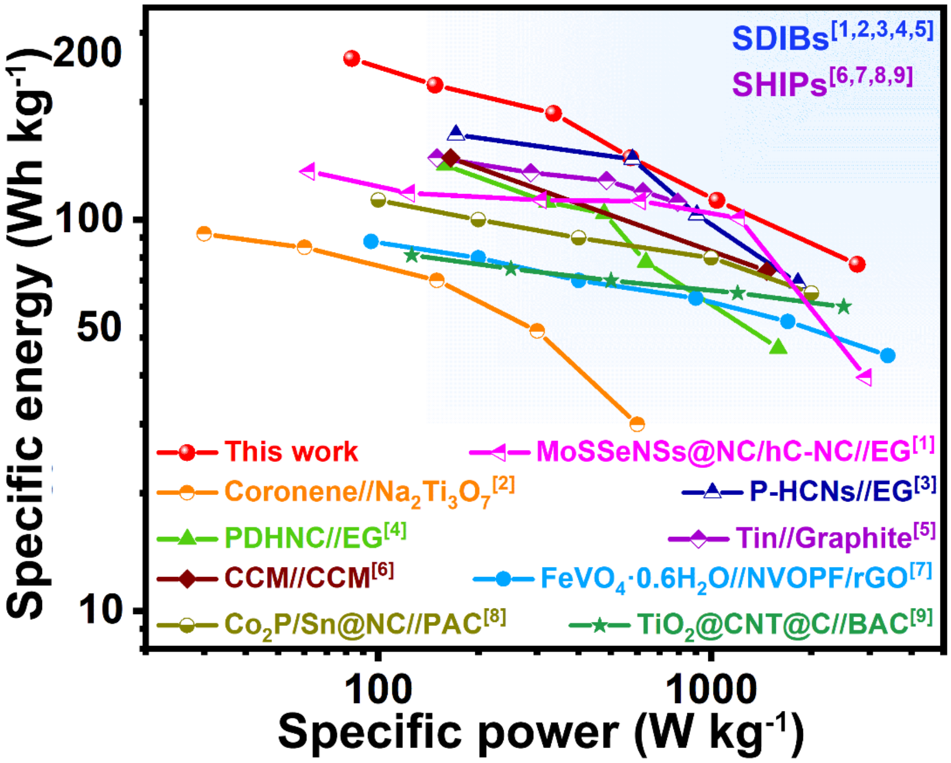

Supplementary Figure 33. Specific energy and powder of our prepared $MoS_{1.5}Te_{0.5}$@C nanocables||EG dual-ion cells in comparison with those of previously reported sodium-based devices (the specific energy and specific power of SDIBs are based on the total mass of both anode and cathode materials).

As shown in Supplementary Figure 33, the $MoS_{1.5}Te_{0.5}$@C nanocables||EG dual-ion cells deliver high specific energy of 192, 183.7, 171.4, 151, 125.5 and 70.1 Wh kg^{-1} at the specific power of 61.9, 123.2, 305, 601, 1168, 2500 W kg^{-1}, respectively.[2-13]

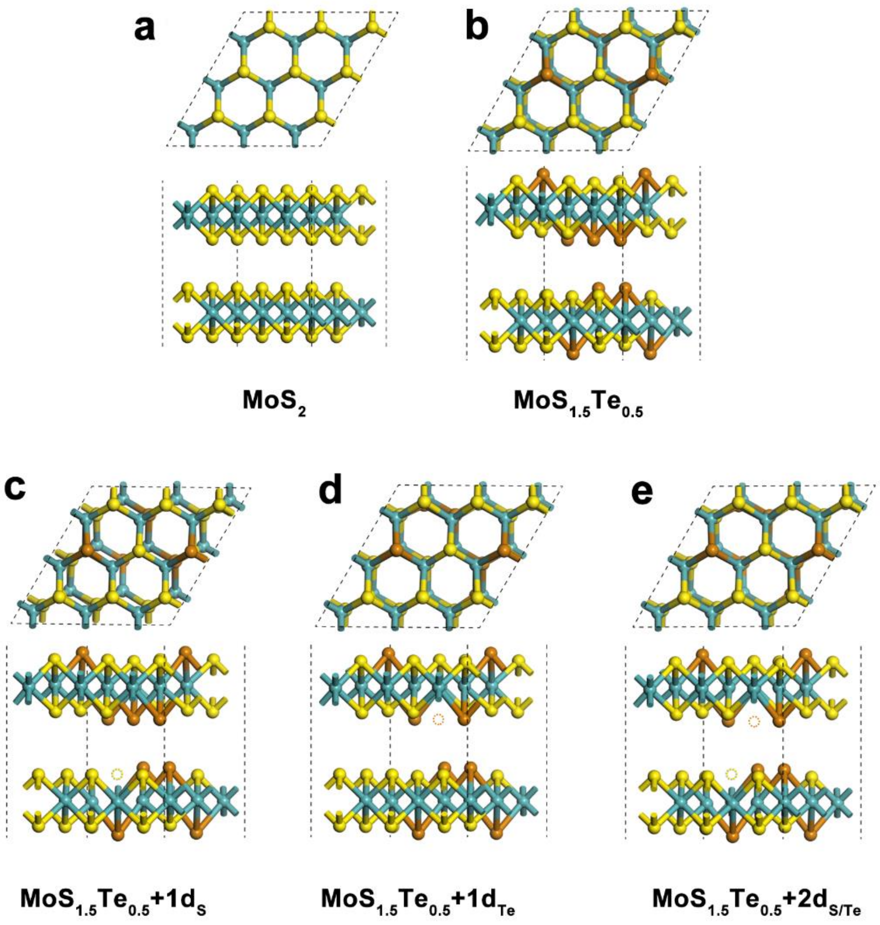

Supplementary Figure 34. Top and side view of the optimized structure of (a) MoS_2 and (b) $MoS_{1.5}Te_{0.5}$, (c) $MoS_{1.5}Te_{0.5}$+1d_S, (d) $MoS_{1.5}Te_{0.5}$+1d_{Te} and (e) $MoS_{1.5}Te_{0.5}$+2$d_{S/Te}$.

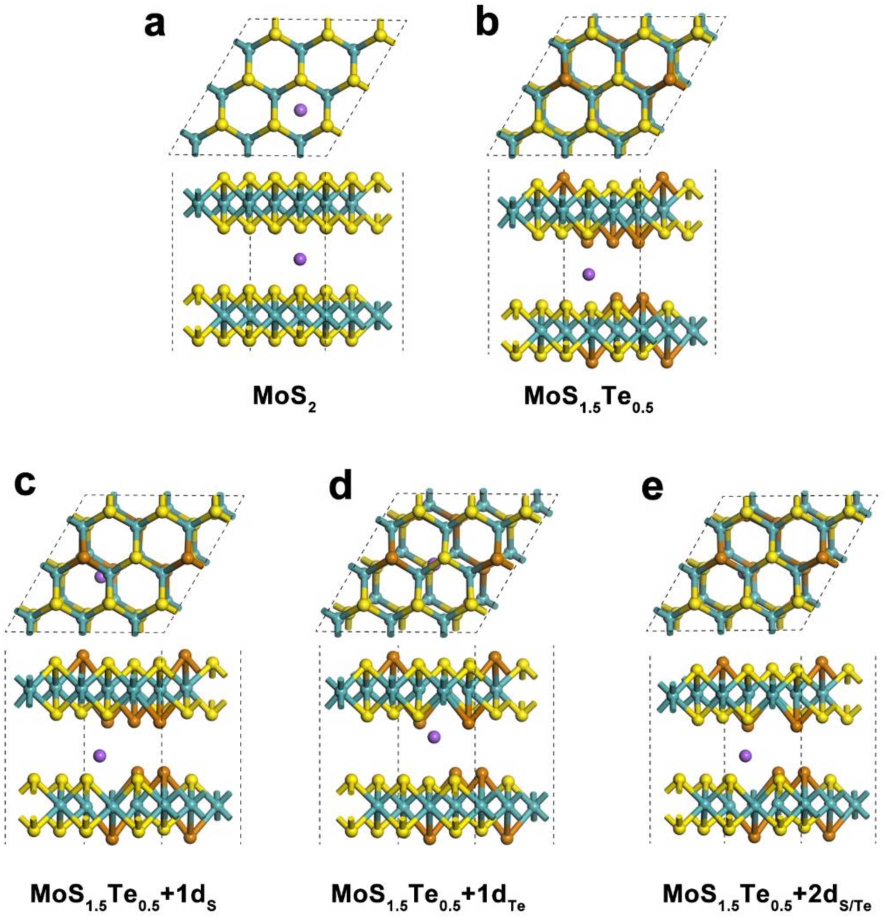

Supplementary Figure 35. Top and side illustration of simulations for one adsorbed Na^+ in the (a) MoS_2 and (b) $MoS_{1.5}Te_{0.5}$, (c) $MoS_{1.5}Te_{0.5}$+1d_S, (d) $MoS_{1.5}Te_{0.5}$+1d_{Te} and (e) $MoS_{1.5}Te_{0.5}$+2$d_{S/Te}$.

The corresponding adsorption energy (ΔE_a) for sodium ions is calculated by adding a single Na^+ in each model and then relaxing the optimized geometry structures again (Supplementary Figure 35), according to the following Supplementary Equation (5):

$$\Delta E_a = E_{tot} - E_{Na} - E_{str} \quad (5)$$

where E_{tot} is the total energy of compound, E_{Na} is the energy of per atom in pure Na metal, and E_{str} is the energy of each structure.

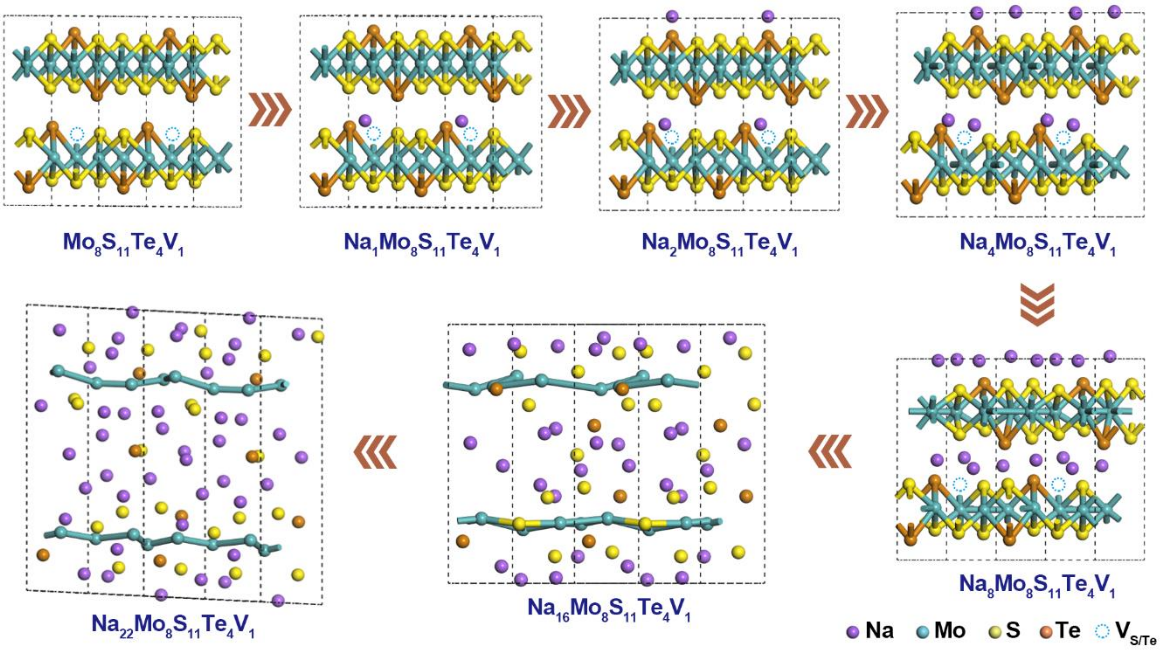

Supplementary Figure 36. Calculated sodiation process of $MoS_{1.5}Te_{0.5}$ by DFT. In sodiation process, the corresponding intermediates are identified by DFT.

As shown in Supplementary Figure 36, several intermediate sodium-ion intercalated phases are predicted and identified according to DFT calculations. During the initial sodiation process, the anion defect sites present stronger electron deficiency characteristics, so that Na^+ tend to insert at anion defect sites, since the anion defects are more likely to gain electrons from Na atoms. The Na^+ intercalated $MoS_{1.5}Te_{0.5}$ lattice would form an intercalated structure when the number of sodium ions is less than or equal to the number of Mo ions. Above the thresholds, Mo ions start to be reduced and the conversion reaction will occur. The final phase after Na^+ insertion includes Mo, Na_2S and Na_2Te.

Supplementary Table 1. Contents of Mo, Te, S, and C in $MoS_{1.5}Te_{0.5}$@C nanocables and $MoS_{1.5}Te_{0.5}$@C nanotubes

Element	$MoS_{1.5}Te_{0.5}$@C nanocables (wt%)	$MoS_{1.5}Te_{0.5}$@C nanotubes (wt%)
Mo[a)]	45.3	43.0
Te[a)]	28.7	27.1
S[b)]	19.8	18.2
C[b)]	9.1	12.8

a) The element results were analyzed by ICP-AES.

b) The element results were analyzed by EA.

Supplementary Table 2. Specific surface area and pore volume of three different Mo-containing active materials

Sample	S_{BET} (m^2g^{-1})	V_{pore} (cm^3g^{-1})
**$MoS_{1.5}Te_{0.5}$@C nanocables**	21.9	0.77
**$MoS_{1.5}Te_{0.5}$@C nanotubes**	24.9	0.091
**CNT@MoS_2 nanotubes**	36.7	0.12

Supplementary Table 3. The error between the raw and fitted data in the equivalent circuit.

	$MoS_{1.5}Te_{0.5}$@C nanocable		$CNT@MoS_2$ nanotube	
**Element**	**Value**	**Error%**	**Value**	**Error%**
R_s	14.75	1.23	19.83	0.94
R_{ct}	90.71	1.49	210.10	2.66
W_1-R	4140	2.55	12311	5.67
W_1-T	1.80	4.35	30.17	7.70
W_1-P	0.69	0.84	0.75	0.50
CPE-T	2.54E-5	8.05	4.59E-5	7.69
CPE-P	0.79	1.15	0.705	1.20

Supplementary Table 4. The EIS fitting parameters of the $MoS_{1.5}Te_{0.5}$@C nanocables and the $CNT@MoS_2$ electrodes.

Sample	R_{ct} (Ohm)	Error (%)
**$MoS_{1.5}Te_{0.5}$@C nanocables**	90.7	1.23
**$CNT@MoS_2$ nanotubes**	210.1	1.06

Supplementary Table 5. Cycle performance comparison of $MoS_{1.5}Te_{0.5}$@C nanocables||EG dual-ion cells with various recently reported dual-ion cells (The capacities were calculated based on the mass of anode).

Materials	Voltage range (V)	Specific current (mA g^{-1})	Cycle number	Specific capacity (mAh g^{-1})	m_{anode}/ $m_{cathode}$ (mg)	The type of electrolytes	Reference
$MoS_{1.5}Te_{0.5}$@C nanocables||EG <u>Na-based dual-ion cells</u>	1.5-4.7	100	100	216.8	1.0/4.0	3.0 M $NaPF_6$ EC/DMC/DEC (1:1:1, v:v:v)	This work
		1000	1500	145.7			
Soft carbon||graphite <u>Na-based dual-ion cells</u>	2.0-4.7	1000	800	54	1.5/2.5-3.0	1 M $NaPF_6$ EC/DMC (6:4, V/V)	(14)
Coronene||$Na_2Ti_3O_7$ <u>Na-based dual-ion cells</u>	1.5-3.5	500	5000	78	0.9/4.5	1 M $NaPF_6$ EC/DMC (1:1, V/V)	(6)
P-doped hollow carbon||EG <u>Na-based dual-ion cells</u>	2.0-4.7	500	1500	121	1.0/4.0	1 M $NaPF_6$ EC/DMC (4:6, V/V)	(7)
N,S-doped MoS_2@C nanosheets||EG <u>Na-based dual-ion cells</u>	1.0-4.0	100	300	60	1.5/2.0	1 M $NaPF_6$ EC/DMC/DEC (1:1:1, v:v:v)	(15)
		1000	5000	39.3			
P-doped soft carbon||graphite <u>Na-based dual-ion cells</u>	2.0-4.7	500	100	110	1.0/3.0	1 M $NaPF_6$ EC/DMC (6:4, v/v)	(16)
		1000	900	81			
Soft carbon nanosheets||EG <u>Na-based dual-ion cells</u>	3.5-4.7	100	350	56.6	1.5/2.0	1 M $NaPF_6$ EC/DMC (6:4, m/m)	(17)
WS_2||graphite <u>Li-based dual-ion cells</u>	0.01-4.0	100	30	47	1.5/6.0	1 M $LiPF_6$ EC/EMC (1:1, v/v)	(18)

Porous N-doped CNF‖graphite K-based dual-ion cells	3.0-5.0	100	346	65	0.5/3.0-4.0	1 M KPF_6 EC/DMC (1:1, m/m)	(19)

Supplementary Table 6. Cycle performance comparison of $MoS_{1.5}Te_{0.5}$@C nanocables‖EG dual-ion cells with various recently reported sodium based dual-ion batteries. (The specific capacity and specific current were calculated based on the total mass of the cathode and anode active materials)

Materials	Voltage range (V)	Specific current (mA g^{-1})	Cycle number	Specific capacity (mAh g^{-1})	m_{anode}/ $m_{cathode}$ (mg)	The type of electrolyte	Reference
$MoS_{1.5}Te_{0.5}$@C nanocables‖EG Na-based dual-ion cells	1.5-4.7	20	100	43.4	1.0/4.0	3.0 M $NaPF_6$ EC/DMC/DEC (1:1:1, v:v:v)	This work
		200	1500	29.2			
Soft carbon‖graphite Na-based dual-ion cells	2.0-4.7	333	800	18	1.5/2.5-3.0	1 M $NaPF_6$ EC/DMC (6:4, v/v)	(14)
Hard carbon‖graphite Na-based dual-ion cells	2.0-4.7	500	200	22.2	1.0/1.8	2.55 M NaTFSI in TMP	(20)
Coronene‖$Na_2Ti_3O_7$ Na-based dual-ion cells	1.5-3.5	100	5000	19.3	0.9/4.5	1 M $NaPF_6$ EC/DMC (1:1, v/v)	(6)
P-doped hollow carbon‖EG Na-based dual-ion cells	2.0-4.7	100	1500	24.2	1.0/4.0	1 M $NaPF_6$ EC/DMC (6:4, m/m)	(7)
N,S-doped MoS_2@C	1.0-4.0	42.9	300	25.7	1.5/2.0	1 M $NaPF_6$ EC/DMC/DEC	(15)

nanosheets‖EG<br>Na-based dual-ion cells		428.5	5000	16.8		(1:1:1, v:v:v)	
P-doped soft carbon‖graphite<br>Na-based dual-ion cells	2.0-4.7	125	100	27.5	1.0/3.0	1 M $NaPF_6$ EC/DMC (6:4, v/v)	(16)
		250	900	20.2			
MoS_2/C-G‖EG<br>Na-based dual-ion cells	1.0-4.0	100	200	27.5	1.0/1.0	1 M $NaPF_6$ EC/DMC/DEC (1:1:1, v:v:v)	(21)
Hard carbon‖graphite<br>Na-based dual-ion cells	1.5-4.8	45	100	21.8	1.0/1.2	1 M $NaClO_4$ PC	(22)

Supplementary Table 7. The calculated values for the ex situ X-ray diffraction measurements of charged PF_6^-EG electrodes of the different cell setups (data corresponding to Figure 5c in in the revised manuscript).

**Cell Setup**	**EG electrodes**
$d_{(n+2)}/d_{(n+1)}$ ratio	1.20
$2\theta_{(n+1)}$ [°]	25.3
$2\theta_{(n+2)}$ [°]	30.5
Dominant stage [n]	3
Periodic repeat distance (I_c) [nm]	1.457
PF_6^- gallery height (d_i) [nm]	0.788
PF_6^- gallery height (Δd) [nm]	0.453

According to the Bragg's Law formula, the graphite layers was expanded to 0.35 nm from the initial value (0.335 nm) when charging voltage to 4.7 V. The determined stages (n) in the formed PF_6^--GIC could be related to the periodic repeating distance (I_c), the gallery height (d_i), and the gallery expansion (Δd), according to the Supplementary Equation (6):

$$I_c = d_i + 0.335\text{ nm} \times (n\text{-}1) = \Delta d + 0.335\text{ nm} \times n = l \times d_{obs} \qquad (6)$$

where l represents the index of (00*l*) oriented in the stacking direction, d_{obs} describes the observed value of the spacing between two graphitic layers, which can be calculated from diffraction angles by Bragg's Law.[23,24]

Based on the above results (Supplementary Table 7), the stage-3 GIC can be formed when the charging voltage approached 4.7 V, implying quite a lot of PF_6^- anions can be stored in graphite space.

Supplementary References

1 Liu, T. et al. A high concentration electrolyte enables superior cycleability and rate capability for high voltage dual graphite battery. *J. Power Sources* **437**, 226942 (2019).

2 Zheng, J. et al. Research progress towards understanding the unique interfaces between concentrated electrolytes and electrodes for energy storage applications. *Adv. Sci.* **4**, 1700032 (2017).

3 Heckmann, A. et al. Towards high-performance dual-graphite batteries using highly concentrated organic electrolytes. *Electrochim. Acta* **260**, 514 (2018).

4 Yamada, Y. et al. Unusual stability of acetonitrile-based superconcentrated electrolytes for fast-charging lithium-ion batteries. *J. Am. Chem. Soc.* **136**, 5039 (2014).

5 Dong, S. et al. A novel coronene//$Na_2Ti_3O_7$ dual-ion battery. *Nano Energy* **40**, 233 (2017).

6 Wang, X. et al. Phosphorus-doped porous hollow carbon nanorods for high-performance sodium based dual-ion battery. *J. Mater. Chem. A* **8**, 4007 (2020).

7 Wang, X. et al. Regulate phosphorus configuration in high P-doped hard carbon as a superanode for sodium storage. *ACS Appl. Mater. Interfaces* **13**, 12059-12068 (2021).

8 Sheng, M. et al. A novel tin-graphite dual-ion battery based on sodium-ion electrolyte with high energy density. *Adv. Energy Mater.* **7**, 1601963 (2017).

9 Wang, X. et al. Commercial carbon molecular sieves as a Na^+-storage anode material in dual-ion batteries. *J. Electrochem. Soc.* **164**, 3649-3656 (2017).

10 Dong, J. et al. Intercalation pseudocapacitance of $FeVO_4 \cdot nH_2O$ nanowires anode for high-energy and high-power sodium-ion capacitor. *Nano Energy* **73**, 104838 (2020).

11 Ren, X. et al. Tailored plum pudding-like Co_2P/Sn encapsulated with carbon nanobox shell as superior anode materials for high-performance sodium-ion capacitors. *Adv. Energy Mater.* **9**, 1900091 (2019).

12 Zhu, Y. et al. Fast sodium storage in TiO_2@CNT@C nanorods for high-performance Na-ion capacitors. *Adv. Energy Mater.* **7**, 1701222 (2017).

13 Liu, B. et al. N-doped carbon modifying MoSSe nanosheets on hollow cubic carbon for high-performance anodes of sodium-based dual-ion batteries. *Adv. Funct Mater.* DOI: 10.1002/adfm.202101066

14 Fan, L. et al. Soft carbon as anode for high-performance sodium-based dual ion full battery. *Adv. Energy Mater.* **7**, 1602778 (2017).

15 Liu, Y. et al. Layer-by-layer stacked nanohybrids of N,S-codoped carbon film modified atomic MoS_2 nanosheets for advanced sodium dual-ion batteries. *J. Mater. Chem. A* **7**, 24271 (2019).

16 Ma, R. et al. Offset initial sodium loss to improve coulombic efficiency and stability of sodium dual ion batteries. *ACS Appl. Mater. Interfaces* **10**, 15751 (2018).

17 Yao, X. et al. Defect-rich soft carbon porous nanosheets for fast and high-capacity sodium-ion storage. *Adv. Energy Mater.* **9**, 1803260 (2018).

18 Bellani, S. et al. WS_2-graphite dual-ion batteries. *Nano Lett.* **18**, 7155 (2018).

19 Zhang, M. et al. Hierarchically porous N-doped carbon fibers as a free-standing anode for high-capacity potassium-based dual-ion battery. *Adv. Energy Mater.* **9**, 1901663 (2019).

20 Jiang, X. et al. A nonflammable Na^+-based dual-carbon battery with low-cost, high voltage, and long cycle life. *Adv. Energy Mater.* **8**, 1802176 (2018)

21 Zhu, H. et al. Penne-like MoS_2/carbon nanocomposite as anode for sodium-ion-based dual-ion battery. *Small* **14**, 1703951 (2018).

22 Hu, Z. et al. All carbon dual ion batteries. *ACS Appl. Mater. Interfaces* **10**, 35978 (2018).

23 Dresselhaus, M. S. et al. Intercalation compounds of graphite, *Advances in physics* **51**, 1-186 (2002).

24 Rüdorff, W. et al. In situ X-ray diffraction studies of cation and anion intercalation into graphitic carbons for electrochemical energy storage applications. *Z. Anorg. Allg. Chem.* **640**, 1996–2006 (2014)
