# Supplementary material for: Carbon-coated MoS1.5Te0.5 nanocables for efficient sodium-ion storage in non-aqueous dual-ion batteries
Source: Nat Commun. 2022 Feb 3;13:663. doi: 10.1038/s41467-022-28176-0 (PMC8814252; doi:10.1038/s41467-022-28176-0)
Supplement: Supplementary file 3 — Description of additional Supplementary File [file 41467_2022_28176_MOESM3_ESM.pdf]

Description of Additional Supplementary Files

File Name: Supplementary Movie 1
Description: Lighting up a "DIB" sign made of 42 light-emitting-diodes (LEDs) using the as-prepared dual-ion cell
