# Supplementary material for: Carbon-coated MoS1.5Te0.5 nanocables for efficient sodium-ion storage in non-aqueous dual-ion batteries
Source: Nat Commun. 2022 Feb 3;13:663. doi: 10.1038/s41467-022-28176-0 (PMC8814252; doi:10.1038/s41467-022-28176-0)
Supplement: Supplementary file 5 — Reporting Summary [file 41467_2022_28176_MOESM5_ESM.pdf]

nature portfolio

Corresponding author(s): Hongbing Zhan; Zhenhai Wen

Last updated by author(s): Dec 29, 2021

Reporting Summary

Nature Portfolio wishes to improve the reproducibility of the work that we publish. This form provides structure for consistency and transparency in reporting. For further information on Nature Portfolio policies, see our Editorial Policies and the Editorial Policy Checklist.

Statistics

For all statistical analyses, confirm that the following items are present in the figure legend, table legend, main text, or Methods section.

n/a	Confirmed	
☒	☐	The exact sample size (*n*) for each experimental group/condition, given as a discrete number and unit of measurement
☒	☐	A statement on whether measurements were taken from distinct samples or whether the same sample was measured repeatedly
☒	☐	The statistical test(s) used AND whether they are one- or two-sided<br>*Only common tests should be described solely by name; describe more complex techniques in the Methods section.*
☒	☐	A description of all covariates tested
☒	☐	A description of any assumptions or corrections, such as tests of normality and adjustment for multiple comparisons
☒	☐	A full description of the statistical parameters including central tendency (e.g. means) or other basic estimates (e.g. regression coefficient) AND variation (e.g. standard deviation) or associated estimates of uncertainty (e.g. confidence intervals)
☒	☐	For null hypothesis testing, the test statistic (e.g. *F*, *t*, *r*) with confidence intervals, effect sizes, degrees of freedom and *P* value noted<br>*Give P values as exact values whenever suitable.*
☒	☐	For Bayesian analysis, information on the choice of priors and Markov chain Monte Carlo settings
☒	☐	For hierarchical and complex designs, identification of the appropriate level for tests and full reporting of outcomes
☒	☐	Estimates of effect sizes (e.g. Cohen's *d*, Pearson's *r*), indicating how they were calculated

Our web collection on statistics for biologists contains articles on many of the points above.

Software and code

Policy information about availability of computer code

Data collection	Quantum ESPRESSO from https://github.com/QEF/q-e/releases
Data analysis	python 3.7

For manuscripts utilizing custom algorithms or software that are central to the research but not yet described in published literature, software must be made available to editors and reviewers. We strongly encourage code deposition in a community repository (e.g. GitHub). See the Nature Portfolio guidelines for submitting code & software for further information.

Data

Policy information about availability of data

All manuscripts must include a data availability statement. This statement should provide the following information, where applicable:

- Accession codes, unique identifiers, or web links for publicly available datasets
- A description of any restrictions on data availability
- For clinical datasets or third party data, please ensure that the statement adheres to our policy

The data that support the plots within this paper and other finding of this study are available from the corresponding author upon reasonable request.

Field-specific reporting

Ecological, evolutionary & environmental sciences study design

All studies must disclose on these points even when the disclosure is negative.

Study description	We here report an innovative and universal source-template synthetic strategy for the fabrication of a variety of nanowire-in-nanotube MSxTey@C nanocables (M= Mo, W, Re), which is implemented by in-situ growth of carbon film modified MS2 nanosheets on the inner and outer surface of tellurium (Te) nanotubes, accompanying with annealing evolution into Te doped MS2. Among these species, the MoS1.5Te0.5@C nanocables are studied as the anode of SDIBs by coupling the expanded graphite cathode in a high salt-content electrolyte (3.0 M), which manifests a prolonged cycling lifespan with 97% capacity retention over 1500 cycles at 1 A g-1 as well as an impressive rate capability with a reversible capacity of 100.9 mAh g-1 under 5 A g-1 based on the mass of the anode.
Research sample	It is no relevant to our study.
Sampling strategy	No sampling size calculation was performed in this study. We will do parallel experiments for each electrochemical testing to ensure sufficient sample-size.
Data collection	We will do parallel experiments and collect data that is close to the average.
Timing and spatial scale	The cell cycling experiments are carried out at climatic chamber (25 °C), which guarantees the consistency of the test environment. The data is collected after corresponding electrochemical tests.
Data exclusions	No data would be excluded from the analyses.
Reproducibility	All attempts to repeat the experiment were successful.
Randomization	It is no relevant to our study.
Blinding	It is no relevant to our study. We tend to collect the electrochemical data that close to the average from a set of data.

Did the study involve field work? ☐ Yes ☒ No

Reporting for specific materials, systems and methods

We require information from authors about some types of materials, experimental systems and methods used in many studies. Here, indicate whether each material, system or method listed is relevant to your study. If you are not sure if a list item applies to your research, read the appropriate section before selecting a response.

Materials & experimental systems

n/a	Involved in the study
☒	☐ Antibodies
☒	☐ Eukaryotic cell lines
☒	☐ Palaeontology and archaeology
☒	☐ Animals and other organisms
☒	☐ Human research participants
☒	☐ Clinical data
☒	☐ Dual use research of concern

Methods

n/a	Involved in the study
☒	☐ ChIP-seq
☒	☐ Flow cytometry
☒	☐ MRI-based neuroimaging
